# Overdose prevention centres as spaces of safety, trust and inclusion: A causal pathway based on a realist review

**DOI:** 10.1111/dar.13908

**Published:** 2024-08-05

**Authors:** Alex Stevens, Jolie R. Keemink, Sam Shirley-Beavan, Zarnie Khadjesari, Adelina Artenie, Peter Vickerman, Mat Southwell, Gillian W. Shorter

**Affiliations:** 1 Social Policy, Sociology and Social Research, University of Kent, Medway, UK; 2 Centre for Health Services Studies, University of Kent, Canterbury, UK; 3 School of Health Sciences, University of East Anglia, Norwich, UK; 4 Bristol Medical School, University of Bristol, Bristol, UK; 5 CoAct, Bath, UK; 6 Drug and Alcohol Research Network, Queen’s University Belfast, Belfast, UK

**Keywords:** drug consumption rooms, harm reduction, overdose prevention centres, realist review

## Abstract

**Issues:**

Overdose prevention centres (OPC) are non-residential spaces where people can use illicit drugs (that they have obtained elsewhere) in the presence of staff who can intervene to prevent and manage any overdoses that occur. Many reviews of OPCs exist but they do not explain how OPCs work.

**Approach:**

We carried out a realist review, using the RAMESES reporting standards. We systematically searched for and then thematically analysed 391 documents that provide information on the contexts, mechanisms and outcomes of OPCs.

**Key Findings:**

Our retroductive analysis identified a causal pathway that highlights the feeling of safety – and the immediate outcome of not dying – as conditions of possibility for the people who use OPCs to build trust and experience social inclusion. The combination of safety, trust and social inclusion that is triggered by OPCs can – depending on the contexts in which they operate – generate other positive outcomes, which may include less risky drug use practices, reductions in blood borne viruses and injection-related infections and wounds, and access to housing. These outcomes are contingent on relevant contexts, including political and legal environments, which differ for women and people from racialised minorities.

**Conclusions:**

OPCs can enable people who live with structural violence and vulnerability to develop feelings of safety and trust that help them stay alive and to build longer term trajectories of social inclusion, with potential to improve other aspects of their health and living conditions.

## 1 Introduction

There are ongoing public health crises of drug-related deaths in the USA, Canada and the UK [1,2]. Such deaths are also a ‘significant public health issue’ in Australia [3], New Zealand [4] and in continental Europe [5]. These deaths are heavily concentrated among groups who suffer from material deprivation, psychological trauma, substance use disorders, co-occurring health problems, physical violence, homelessness and other aspects of extreme social exclusion [6–10]. There is an urgent need to engage vulnerable people, particularly into services that prevent them from dying [2].

Overdose prevention centres (OPC) were first operated in Switzerland and Germany in the mid-1980s [11], then spread to other countries in continental Europe [12], and then to Australia, Canada, Mexico, Colombia, Iceland, Scotland (the UK) and the USA [13]. OPCs are non-residential spaces where people use illicit drugs in the presence of staff who can intervene to prevent and manage any overdoses that occur. OPCs have alternative names such as drug consumption rooms and various others [14]. Here, we use OPC as an umbrella term that covers all such services.^1^

OPCs do not provide the drugs that are consumed in them, but can be in the same neighbourhoods as services that provide prescribed opioids and other forms of ‘safer supply’ [15,16]. They are low-threshold services that deliberately reduce barriers to access. They are generally open to people from all social backgrounds, but are pre-dominantly used by people who face various forms of social exclusion, including homelessness or unstable housing [17–22]. OPCs can offer facilities for use of drugs by injection or inhalation, and can also provide a range of other services, including advice on injecting technique, care for injection-related wounds and infections, checking of drug contents, access to primary health care, and onward referral to housing and drug treatment services [23].

There are already several systematic reviews that cover the outcomes of OPCs [24–30]. They report on a range of outcomes, generally finding that OPCs have a positive impact in reducing and reversing overdoses and injecting risk behaviours, increasing uptake of drug treatment services, with little or no impact on crime [11,31]. There have also been three reviews of the findings of qualitative research on OPCs [32–34]. The provision of OPCs has recently been recommended by both the European Monitoring Centre for Drugs and Drug Addiction and the European Centre for Disease Prevention and Control ‘in order to reduce injecting risk behaviour among people who inject drugs’ [35].

Here, we report on the first realist review of OPCs to reveal the underlying mechanisms and complex causation of their effects [36]. We aim to synthesise evidence from multiple sources to theorise the causal pathway by which components of OPCs combine with their contexts and mechanisms to produce outcomes [37,38]. Unlike a traditional systematic review, a realist review aims to understand how an intervention works, not just if it works. It incorporates some of the criteria used by Austin Bradford Hill to identify causal relationships (e.g. his interest in the plausibility and coherence of proposed causes), but goes further to make inferences about real causal mechanisms which underly the statistical associations and dose responses which he looked for [39].

Realist reviews often including a wider range of research methods and studies, including observational and qualitative research, and not just the randomised controlled trials on which systematic reviews tend to concentrate [36,40]. The critical realist assumption is that practically adequate knowledge is to be gained by inferring the underlying generative mechanisms of a complex intervention, not just by looking for constant conjunctions of independent and dependent variables in experimental and quasi-experimental research [41–43]. For OPCs in particular, a realist understanding of the contexts, mechanisms and outcomes may help to inform the development and evaluation of new services in response to the ongoing crises of opioid deaths in several countries. OPCs were originally developed to meet the needs of people who inject heroin. As drug markets develop, with wider use and availability of potent synthetic opioids by both injecting and inhalations, it will be useful to understand how OPCs work for particular groups in particular settings.

This article reports on our realist review to answer the question: how can we explain the outcomes that have been observed in studies of OPCs? We include specific examination of how the identified causal pathway works for particularly vulnerable groups, including women and people from racialised minorities.

## 2 Methods Of The Realist Review

The protocol for this review was registered in PROSPERO (CRD42023414273) [44] and the review is reported using RAMESES reporting standards [45]. We first built an initial program theory on existing reviews and through consultation with stakeholders in the field.^2^ These included members of the project advisory board, members of the Drug Science Enhanced Harm Reduction Working Group, and representatives of people who use drugs, including members of the European Network of People who Use Drugs. We provide a visual representation of this initial program theory in Appendix S1.

From these reviews and consultations, we also created a list of search terms, as shown in Table 1. We used these search terms in the bibliographic databases PubMed, Scopus and the Web of Science. We also searched in the database of grey literature of the International Society for the Study of Drug Policy and the references used in a recent narrative review [46]. Our search was limited to documents published in English, although many of these included insights from studies published in other languages, or were themselves translated from other languages.

We screened titles and abstracts, using the software application Rayyan. Ten percent of the identified documents were screened by two researchers (JK and AS), to agree the process for inclusion and exclusion. We then downloaded full versions of the documents into a Zotero library which we then uploaded into NVivo for analysis. We excluded documents that did not meet inclusion criteria, and included cited documents that were referred to in the selected documents where they met criteria. We included studies that provided data about the operation of actual OPCs (not just proposed services). There were no time limits, however, the earliest record we found was published in 1999.

We extracted data from the included documents by highlighting segments of text that were relevant to the contexts, mechanisms and outcomes of OPCs in Nvivo [47]. We follow Greenhalgh and Manzano in thinking of contexts as layered, relational and dynamic features of the environments within which OPCs operate that affect how it works [48]. These include some contexts that pre-exist the operation of the OPC, while others emerge from the interaction between the interventions provided by the OPC and its environment. The latter are described below as ‘dynamic contexts’. We understand mechanisms as the underlying causal processes which are triggered by the various components of OPCs in their contexts and which generate the outcomes of OPCs [49].

In Nvivo, we added codes to those based on the initial program theory as we found other relevant concepts in the documents we reviewed. We then reorganised these provisional and emergent codes into core and satellite concepts. In this way, our process was compatible with both adaptive and abductive analysis [50,51]. The development of the causal pathway presented here was an iterative process of reading, coding, re-reading and re-coding the selected texts in discussions between the research team. There were many intermediate stages of analytical development between the initial program theory shown in Appendix S1 and the causal pathway presented in Figure 2.

In carrying out this analysis, we drew on Tim Rhodes’ concept of the ‘risk environment’ to think about the socio-economic contexts in which OPCs operate [52]. We used the first two levels (physiological and safety needs) of Abraham Maslow’s well-known hierarchy of needs to think about how OPCs may help people to satisfy their needs [53]. We used the COM-B model from Michie, Atkins and West’s explanation of how capacity, opportunity and motivation combine to produce behavioural change [54]. Our critical realist approach to this review is based on the ontological assumptions of critical realism, which include that the actual phenomena that are available for examination are caused by real, underlying generative mechanisms which cannot be directly observed, but can be inferred from the traces they leave in empirical reality [42,55].

Retroduction is the process by which these mechanisms are inferred [51]. This was the final stage of our analysis. This is an interpretive form of inference that moves from empirical observations of actual events to theorise the underlying generative structures [49,51]. This inference must go beyond the empirical evidence on observed events to suggest provisional conclusions on underlying, contingent combinations of context, mechanism and outcome. It asks: what makes the outcome of an intervention possible? In this way, retroduction identifies the theorised causal pathways by which interventions lead to outcomes. It attempts to identify the essential conditions of possibility of outcomes, so reducing some of the apparent complexity of the phenomenon (this is why the causal pathway presented here contains fewer items than our initial program theory). To summarise such pathways, we state if [the necessary combination is present] then [the outcome will usually occur] because [a generative mechanism or mechanisms is/are triggered] [56,57].

Given the remaining complexity of the social world, any strict division between contexts, mechanisms and outcomes is bound to blur at some points in the causal process [58]. For example, an outcome that is triggered by one mechanism may go on to form the context or trigger for another mechanism, which leads to another. We try to capture some of this complexity in a causal path diagram (Figure 2).

The research involved no primary data collection and so required no ethical approval.

## 3 Results

### 3.1 Documents selected

**Figure 1:**
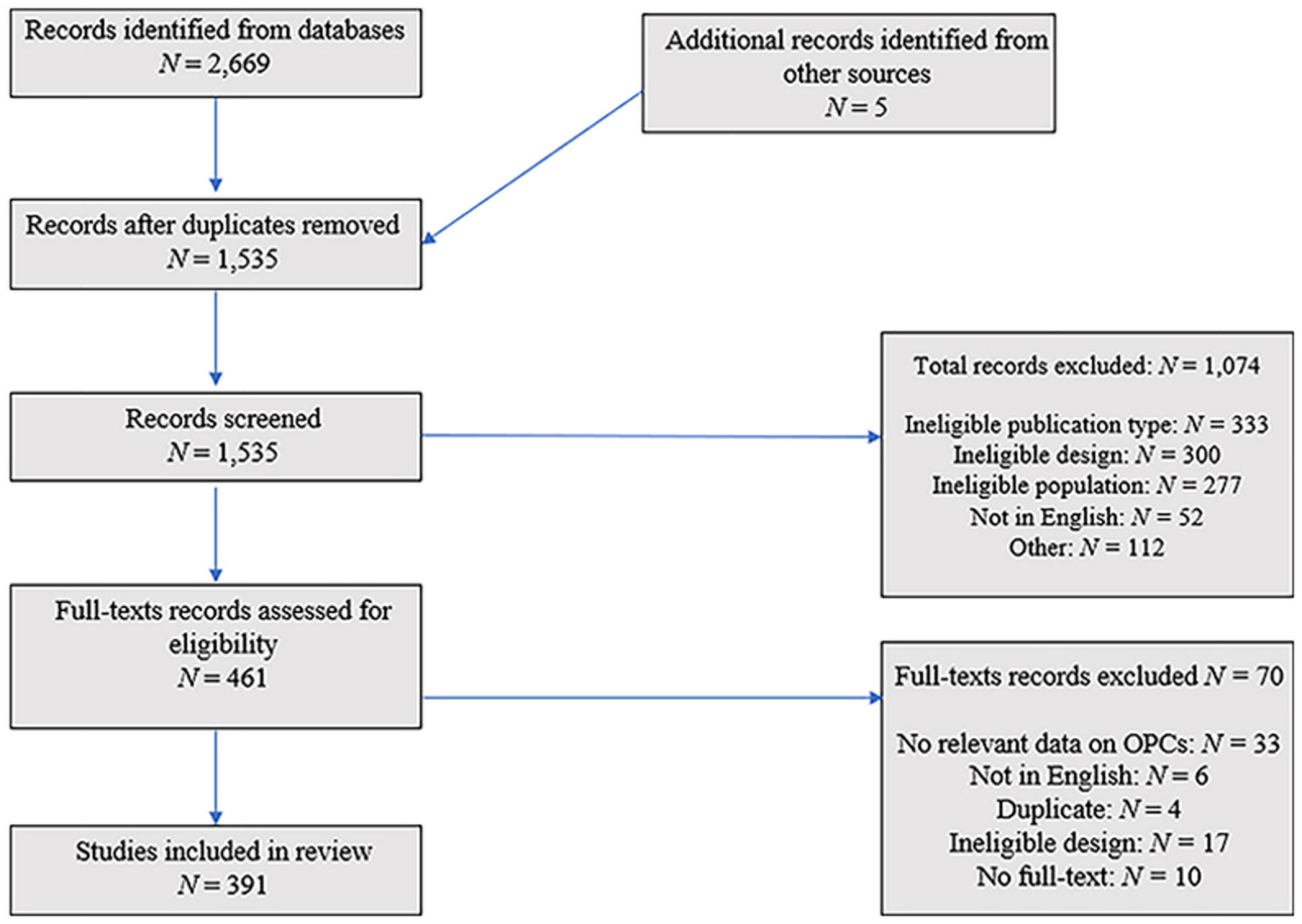
PRISMA diagram of document selection.

We present the results of the literature search and document selection in the PRISMA diagram [59] in Figure 1, including reasons for exclusion of 1144 documents from our final dataset of 1535 articles and reports. Realist reviews take a different approach to document selection than most systematic reviews, which tend to focus on particular criteria for methodological quality [60]. Our reasons for inclusion rather mirrored Dada et al.’s suggestion of focusing on documents that provide relevant, rich and rigorous information to inform the development of realist theory [40]. Documents coded as ‘ineligible publication type’ included commentary and discussion pieces, which could not provide rich data. Documents coded as ‘ineligible design’ included feasibility studies of OPCs that did not actually operate, and so were not considered relevant. Documents coded as ‘ineligible population’ included studies that did not report data on OPCs, but only on other, less relevant services. Documents coded as ‘other’ included, for example, conference abstracts which did not provide empirical data. We have included comments on the rigour of the included studies, where necessary.

Included documents reported on OPCs using a variety of research methods, as displayed in Table 2. Several documents used more than one research method. The studies were heavily concentrated on OPCs in Canada and 89 of them reported findings from one OPC; Insite in Vancouver.

The selected documents also included information on 88 other OPCs in 17 countries, as listed in Table 3. This did not include all actually operating OPCs. In 2018, the European Monitoring Centre on Drugs and Drug Addiction reported that ‘there are: 31 facilities in 25 cities in the Netherlands; 24 in 15 cities in Germany; five in four cities in Denmark, 13 in seven cities in Spain; two in two cities in Norway; two in two cities in France; one in Luxembourg; and 12 in eight cities in Switzerland’ [61]. There is also an OPC that opened in Bogot,a, Colombia in 2023.

Not all OPCs covered by the selected documents are still operating. For example, the three reported in Australia include the temporary ‘tolerance room’ that preceded the opening of the Sydney Medically Supervised Injecting Centre (MSIC) [62]. The OPC in the United Kingdom was an unsanctioned service that operated in Glasgow only in 2020/21 [63]. Unsanctioned services operate with no official, governmental permission or funding and so are more likely to be temporary.

Supporting Information includes a list of the selected documents (Appendix S2) and a list of the OPCs they cover in each country (Appendix S3).

### 3.2 The main causal pathway of OPC contexts, mechanisms and outcomes

**Figure 2:**
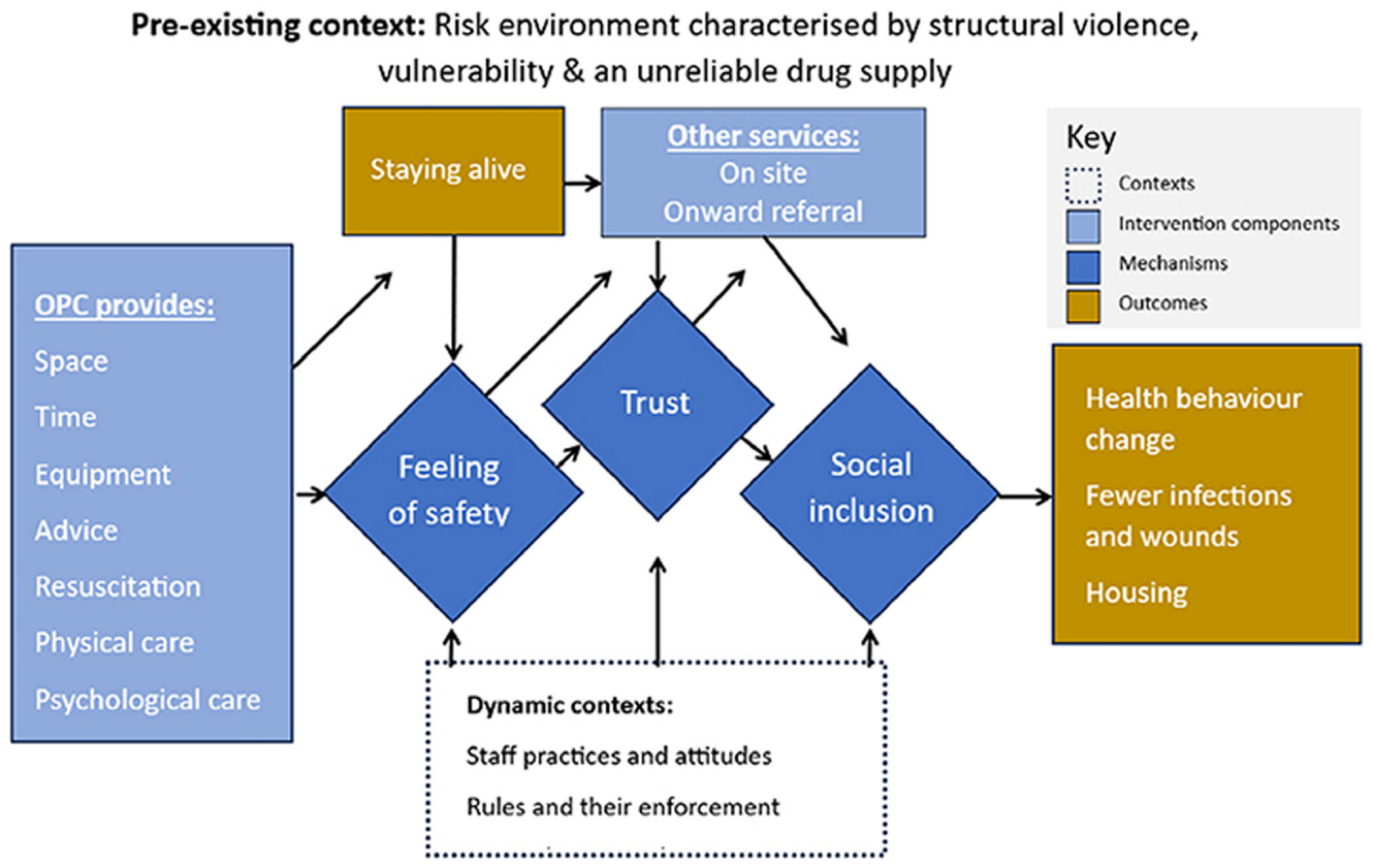
Causal pathway diagram for overdose prevention centres (OPC).

To illustrate the main causal pathway identified in our retroductive analysis, we present it as a diagram in Figure 2. This diagram shows the schematic connections between intervention components that are provided in specific contexts which trigger particular mechanisms and outcomes.

The causal path that is illustrated in this diagram is based on our realist synthesis of data from OPCs between which there are large differences in terms of the drugs being used, the legal and drug policy contexts, the neighbourhoods they are based in, their cultural environment, and the social and health systems available to the people who attend OPCs. These form the ‘risk environment’ for people who are involved in street-based drug use [52]. This may be very different – for example – between a setting like New York City (where two OPCs recently opened in largely Hispanic neighbourhoods, with high levels of street homelessness, HIV, and limited access to high quality health and welfare services, in a drug market saturated with fentanyl, and in a precarious legal environment for harm reduction services) and a setting like Geneva (where the Quai 9 OPC has long operated within an eco-system of relatively strong health and social support, which includes access to social housing, opioid agonist therapy – including heroin-assisted treatment – and drug checking services, where heroin is still considered the most problematic drug, and harm reduction has firm institutional support) [64,65]. This is hugely influential on the levels of vulnerability that are experienced by the people who use OPCs.

### 3.3 The socially structured contexts of risk and vulnerability

The experience of structural violence and vulnerability described by Rhodes et al [52] was evident in many of the studies we reviewed, with the risks of violence and rapidly changing drug markets added to the pre-existing contexts of OPCs in many of their locations. People who use OPCs are typically exposed to very high levels of homelessness, violent victimisation, trauma and material deprivation [66–69]. Even in settings with relatively strong health and welfare systems, people who have drug problems tend to be the most marginalised and victimised in their communities. These issues may be particularly acute for women, those with marginalised gender identities, and members of racially marginalised groups, including Indigenous people [70,71]. They are more commonly reported for people who use OPCs than for other people who use the same drugs. For example, a study of young people who injected heroin in Spain found that those who used OPCs were even more vulnerable than those who did not, with higher levels of homelessness and illicit income [17]. In Vancouver, homelessness and public drug use were predictive not only of willingness to use but also of actual use of OPCs in a cohort of people who inject drugs [18]. In Ottawa, a survey of people who inject drugs or smoked crack cocaine found that – of those who were willing to use an OPC – 60% were unstably housed, 50% had their movement restricted by law enforcement agencies and 13% were HIV positive [72].

As drug markets change, with the arrival of potent synthetic opioids, people who are involved in street-based injecting become even more vulnerable to overdose and death. Their awareness of this varies across population groups. One US study of young users of prescription opioids found low levels of perceived risk, even among those who had previous experience of overdosed [73]. The socially structured aspects of this vulnerability are observed in the criminalisation and displacement of people who use drugs [74–76], legal restrictions on the provision of harm reduction services [24,77,78], and decisions to restrict access to basic services. See, for example, the link between the reduction in provision of supported housing for people with mental health problems in Vancouver and the increased number of people involved in street-based injecting in the city in the 2000s [79].

Conversely, improvements in housing provision for people who use drugs in the Netherlands has been associated with a reduction in demand and even closure of some OPCs [23]. In contrast to the Dutch experience, the number of people who inject drugs in North America has substantially increased [80,81], and their environment has been made dramatically riskier by the entry of highly potent synthetic opioids, including fentanyl, into the illicit market [82].

### 3.4 Mechanisms of safety and staying alive

When Maslow developed his hierarchy, he did not consider the need for drugs as a basic physiological needs. However, for some people, use of a substance on which they have become dependent – and so avoiding the onset of physical withdrawal symptoms – can be felt as their most urgent need [83,84]. OPCs do not meet this need by supplying substances to consume but can solve the problem of space to use drugs, when they are open. Outside these places and times, studies in multiple countries have reported high levels of drug use in public in some urban areas, with associated problems of discarded paraphernalia and riskier injecting practices, including rushed injecting with non-sterile water and equipment [85–91]. Space and time are important contexts for the creation of safety for people who are involved in street-based drug use. Using in public exposes people to the public gaze and risk of police detection. Both are experienced as stigmatising and harmful [67,74]. Some people have reported using in public because it is safer for them [88]. They may fear dying if they over-dose alone in a private setting, with nobody there to revive them. The reality of these fears is confirmed by a previous review which found that public injecting is associated with the risk of overdose, and linked to the need to consume hastily to avoid being seen, interrupted or arrested [92].

In contrast, OPCs can provide not only a space in which to use drugs, but also time to do so more safely and comfortably, sterile injecting equipment and advice on how to use it more safely, and if overdose occurs they can be managed using oxygen and naloxone if necessary [93]. Various other forms of psychological and physical care can also be provided.

These may include a friendly welcome, a place to be warm and dry, food, drink and cleaning facilities, as well as more clinical support [94–96]. In contexts where the supply of illicit drugs contains highly potent synthetic opioids, OPCs can provide information which people can use to reduce the risks they run by checking the contents of their drugs [75,97–101]; information which drug sellers may also use to reduce risks to their customers [102]. Most of the studies of OPC provision of drug checking are from Canada. Such services have also been provided at OPCs in Australia and Denmark, but have different effects where there is lower presence of fentanyl, as in most illicit drug markets outside North America [23,103,104]. Drug checking can be provided by using fentanyl testing strips, as is done at several OPCs in North America [98]. In Europe, it is more common for drug checking services to use more reliable and expensive methods that involve spectrometry or chromatography, usually at sites that are physically separate from OPCs [105].

While OPCs do not meet the physiological need for drugs, they can provide the second level of Maslow’s hierarchy of needs, which is safety. In the terms of Michie et al., this is indicative of physical opportunity (a safe environment) and social opportunity (a supportive group of people) which can support positive behaviour changes [54]. The operation of OPCs as places of safety is a recurrent theme in qualitative research from multiple countries and locations [28,32–34,66,67,70,95,106–132]. This includes safety from overdose, but also from infection transmission, police detection and arrest, public stigmatisation and violent victimisation. Many people who use OPCs have reported to researchers that one of the things that makes them feel unsafe is their exposure to being policed while using drugs on the street, and how this incentivises rushed and risky drug use practices. OPCs reduce these people’s exposure to being directly harmed by arrest and criminalisation, as well as incentives to use in risky places and ways.

Physical violence operates alongside the criminalisation of people who use drugs to shape the environment outside OPCs. These services are experienced as spaces of refuge from this risk environment. A man who used an OPC in Frankfurt summed up this feeling of safety in a quote:

‘Out on the streets you’re always under pressure and have this fear that the police are going to catch you. Or you’re in the toilet and someone knocks and yeah, you’re in a rush. You can’t enjoy your kick. That’s the problem. And here you have your peace. You, you’re safe.’ [133]

It is interesting that this quote is from one of the few studies of OPCs that directly addresses the pleasure of drug use, and how the environment provided in the OPC can affect it. This has also been studied in La Sala in Barcelona, and SisterSpace in Vancouver, as well as La Strada in Frankfurt [114,134,135].

The provision by OPCs of clean space and sterile equipment for drug use means that drug use is more hygienic in OPCs than it would be outside. Such services can also have effects beyond the OPC. For example, advice provided by OPC staff on how to use drugs more safely (such as safer injecting techniques or improved hygiene) may affect the safety of drug use that takes place outside the OPC [65,135,136]. However, Houborg and Jauffret-Roustide note that conceptions of safety reported by people who use OPCs go beyond the narrower hygienic meaning often used in discussions of public health [118]. Safety involves refuge, respite and peace from various experiences of structural violence, as well as reduced risk of overdose deaths and blood-borne viruses.

The need to feel safe was reported as a key motivation for people to use an unsanctioned OPC in Toronto; one described this service as ‘our safe sanctuary’ [107]. Maslow’s is not the only psychological framework to suggest that people’s basic needs – including safety – must be fulfilled before they can address other common needs [133,137]. Here, we suggest that this feeling of safety is a condition of possibility for the generation of positive outcomes from OPCs. Without safety, people may avoid using these services, as was observed when a mobile overdose prevention site was perceived to be less safe than the larger supervised consumption site which it replaced in Lethbridge, Canada [117].

### 3.5 Staying alive

The most immediate outcome experienced by people who use OPCs is that they do not die. People who use OPCs are frequently quoted as stating that the OPC ‘saved my life’ [120,125,128,132,138]. There is even an OPC in Hamburg which is called ‘Stay Alive’ [139]. Many thousands of overdoses are reported as having been reversed by OPCs providing first aid, oxygen and naloxone when needed. This includes over 10,000 overdoses reversed in 21 years of operation at the Sydney MSIC [140]. In all the years and places that have had OPCs in operation, we found reports of only three deaths; two in Germany, and one in the Netherlands [11,94,141]. Only one of these was reported as an overdose and this happened in a toilet in the OPC, rather than in the room designated for drug use.

Two systematic reviews of quantitative studies suggest that OPCs reduce mortality among people who use them [25,26]. The most widely cited primary study of the effect of OPCs on mortality showed that deaths reduced more (by 35%) in the immediate vicinity of the first officially sanctioned OPC in Canada than in neighbouring parts of Vancouver (where such deaths reduced by 9% in the same period) [142]. Other Canadian studies also suggest reductions in death. For example, Kennedy et al.’s study of a cohort of people who inject drugs in Vancouver found lower rates of all-cause mortality among those who were frequent users of an OPC, even when controlling for potentially confounding variables, with an adjusted hazard ratio of dying of 0.46 for these frequent OPC users [143].^3^

Several studies that did not directly examine effects on deaths have shown reductions in strong indicators of the risk of dying, such as non-fatal overdoses and ambulance call outs to overdoses [138,144,145]. However, some studies that have looked for effects on mortality did not find them [146,147]. This may be an artefact of the relatively low number of deaths, compared to other outcomes. For example, early evaluation of the Sydney MSIC found an effect in reducing ambulance call-outs (a more common outcome), but not deaths (which the study had less statistical power to detect) [148]. A later study estimated that this OPC prevented between 55 and 110 deaths between 2007 and 2014 [149]. Other modelling studies have also estimated reductions in deaths from OPCs [150,151]. None of the reviewed studies found that OPCs increase deaths. However, there may be some configurations of context and mechanisms (e.g. limited capacity and opening hours, failure to provide feelings of safety and trust) that prevent OPCs from saving lives, as has been reported in the case of the Lethbridge overdose prevention site [117].

The placement of outcome of staying alive in Figure 2 is an example of the complexity of the causal pathway we identify. The immediate outcome of staying alive that results from using drugs in an OPC then becomes a trigger for the mechanism of feeling safe. This, in combination with other OPC components and mechanisms leads to other outcomes in addition to staying alive.

### 3.6 Mechanisms of trust and social inclusion

Our theorised causal pathway suggests that creating a feeling of safety and actually saving lives, combined with the various services that OPCs provide and refer to, trigger the mechanisms of trust and social inclusion.

Trust is an important mechanism that helps people work with each other towards shared goals [152]. Without trust for the OPC and its staff, people are unlikely to use it [76,153]. Building trust then helps people to make connections with other people and services [67,99,112,119,125,154]. Many of the people who use OPCs have low levels of trust in main-stream healthcare providers. For example, a study of an OPC in Barcelona reported the case of a man who had been diagnosed with hepatitis C, but did not believe it until this was confirmed by someone he knew at the OPC. He said, ‘I don’t ask doctors; I ask people I trust’ [135]. A Canadian study reported that ‘many participants stated this was the first time they had formed a trusting, meaningful connection to a health or social service provider’ [128].

Social inclusion is ‘the process of improving the ability, opportunity, and dignity of those disadvantaged on the basis of their identity, to take part in society’ [155]. In this framing, the process of social inclusion depends on people having access to resources, services and spaces. OPCs can provide all three, but only if people feel safe enough to use them; another example of the complex interdependence of mechanisms and outcomes.

The documents we reviewed provided many examples of OPCs providing spaces for people to change their actions and opportunities through their inclusion in networks of support. Qualitative studies of OPCs repeatedly show that they are places where people can find community, camaraderie and mutual assistance [70,117,121,128,129,136]. Feeling safe and trusting the OPC provides a platform for making helpful connections. These can be to healthcare services that are directly related to drug use, including vaccination, and testing and treatment for blood-borne viruses [156–159]. Other primary health services can also be provided, including distribution of condoms and sexual health information, dentistry, and tobacco smoking cessation [140,160–163]. Access to drug detoxification and treatment is often facilitated by OPCs, whether on-site [112,125,164] or by onward referral [109,156]. This wide range of services can create significant benefits for individual and public health.

As social inclusion is a process, and not a static outcome, different people will experience different benefits, depending on their own interactions with the components, contexts and mechanisms of OPCs and their environments, at different times. Some people who begin to use OPCs are highly socially excluded, and the OPC may be the only service they engage with. There is great heterogeneity of use of OPCs. Some people use them frequently over long periods, while other visit infrequently [65,163]. For some, the process of social inclusion may be limited to having a safe place to get off the street, sterile equipment and a booth to use drugs in, and a friendly welcome and goodbye. We also found reports of longer term and deeper engagement with OPCs and the services they refer people to triggering greater reductions in drug-related harm and exposure to structural violence [30,165].

The outcomes we include in Figure 2 are not intended to work as outcome measures for OPCs. For example, the causal path from OPC provision to housing is hugely contingent on the presence of enough homes for people to live in, and suitable support for people with mental health and drug problems to stay housed. Rather, our proposed causal pathway suggests that, with the right combination of access and support, OPCs can form part of the pathway that takes people from positions of unstable housing and extreme risk to places where they are safer.

### 3.7 Dynamic interactions between contexts and mechanisms

The staffing and practices of OPCs act as dynamic contexts of these mechanisms of safety, trust and social inclusion. These influential contexts emerge in the interactions between the settings and staff of OPCs and the people who use them. The enforcement of tight rules and limited opening times can exclude potential users [65,70,76,95,140,166]. For example, banning assisted injecting (which is illegal in some jurisdictions) or injecting into the jugular vein (which is considered particularly unsafe) excludes people who cannot inject themselves, or have no other veins left to use [126,167,168]. The differing services that OPCs provide – such as access to various forms of drug checking – will mediate the level of safety they can provide to their users. This is another example of how the wide variety of services that can be provided at or near OPCs will have effects on the mechanisms and outcomes that they trigger.

Access and trust can be boosted by the presence of people who have direct experience of drug use in the staff team [33,99]. The balance between accessibility and legality was observed, for example, at an unsanctioned OPC in Italy that was open 24 hours a day. Occasions of use of the OPC for illicit purposes (e.g. stripping copper from stolen electronic equipment) were reported, but the extended opening hours also enabled the OPC to provide naloxone to reverse overdoses that happened at night [169].

Another context that may affect the triggering of social inclusion is the physical layout of services that surround OPCs. In particular, there are substantial differences in ease of access to additional services and connections which depend on whether these are provided at the same site. Many OPCs in Europe are colocated with other services, including needle and syringe programs [61,109]. A survey in the Netherlands, for example, found OPCs that are co-located with ‘living rooms’ for people to rest and relax, overnight accommodation, opioid agonist treatment, advice on budgeting, specialist medical consultations, access to computers, alcohol consumption spaces and heroin-assisted treatment [170]. In Canada, a distinction is drawn between very low threshold overdose prevention sites, and supervised injecting facilities that offer a wider range of services and more highly trained medical staff [14]. The Insite supervised injecting facility in Vancouver has had a co-located ‘Onsite’ drug detoxification service since 2007, enabling direct access to treatment [164]. A study of the relative advantages and disadvantages of these models in Toronto suggests that integrated services can provide ‘convenience and access to other health and social services’, but may also have ‘negative consequences … including building design, lack of privacy and anonymity, and limited hours of operation’ [171]. In Sydney, the proximity of the MSIC to a nearby primary heath care centre for people who inject drugs was reported to facilitate access to these health services [172]. However, in Melbourne, some users of the medically supervised injecting room reported a preference to access other services elsewhere [163]. Such differences in service provision and user preference will have different effects on the triggering of social inclusion for different people.

### 3.8 Contingent outcomes of inclusion

Social inclusion can generate growing beliefs about capabilities for change, and so to positive outcomes [54]. In our review, we found reports of positive effects on numerous outcomes besides mortality, including reduced risk behaviours for the transmission of blood-borne viruses [25,110,148,173–176], better care for cutaneous injection-related infections and wounds [107,177], reduced use of emergency medical services [99,109,140,146,154,163,178–181], and reductions in unsafe disposal of injecting equipment [148,169,174,176,182,183]. Some studies reported that people gained control over their drug use, with some reducing or ending injecting drug use, or stopping illicit drug use altogether [184–187]. There are also several reports of people finding housing through OPCs, although this effect has not been systematically studied [34,95,117,121,132,139].

Most of the studies that have looked at the economics of providing OPCs have used estimates rather than actual data on effects, and generally found positive returns on investment [148,188–191]. Two studies estimated that Insite saved more money than it cost to provide [192,193]. A study using actual data from an OPC in Calgary suggested that the costs saved by avoided emergency health service use were large enough to outweigh the cost of providing the OPC, even without considering the avoided costs of deaths. [180] However, this study may have lacked rigour in that it assumed that every overdose that occurred in the OPC would otherwise have led to an emergency visit, which may not have been the case.

These outcomes are not universally produced by every OPC. For example, a study from Catalonia found large reductions in public injecting among users of an OPC, and increases in safe syringe disposal and entry to drug treatment services, but it did not find a difference in non-fatal overdoses or drug use, reflecting other findings on continued drug use by users of other OPCs [20,94,106]. In Lisbon, a study of community perceptions of the city’s first mobile OPC found a reduction in the visibility of public injecting, although concern about street crime and discarded injecting equipment remained high [194]. In France, people who had access to either of the OPCs (in Paris and Strasbourg) were less likely to share injecting equipment than those (in Bordeaux and Marseille) who did not, but significant differences were not found for HCV testing or in use of opioid agonist therapy [195]. Neither did a time series analysis from the early years of the Sydney MSIC find a reduction in hepatitis C infections [148,175].

This exemplifies how the effects of OPCs are contingent on the political, local and individual contexts within which they operate. These contexts may prevent the operation of helpful mechanisms, and may even trigger harmful mechanisms and events. In many places, OPCs have only been opened after long political struggles, sometimes as acts of civil disobedience [62,63,69,79,107,126,136,169,196–198]. The pre-existing political climate goes on to affect the dynamic contexts of the operation of OPCs, including the limitations on accessibility and service provision which we have mentioned above.

### 3.9 Complex contingencies of gender and race

These complex interactions of contexts and mechanisms can lead to particular effects for specific groups of people who use drugs [199]. Some studies draw attention to the gendered and racialised aspects of the operation of OPCs [70,97,99,111,113,200]. It is worth examining how OPCs work in particular ways for these groups, so as to inform the development of services that are sensitive to issues of gender and racialisation.

Safety may be an especially important mechanism for women, including trans women. A Canadian woman who used an OPC reported, “It’s like a little space of comfort, surrounded by chaos and stigma and hiding and paranoia.” The same study reported a trans woman being attacked and advised to hide her gender identity in the OPC [70]. For women and trans people who are exposed to high levels of gender-based violence, their access to OPCs is mediated by the presence of violent men at the OPC. They may be less willing to use these services [201] but at least one study has found higher willingness to use an OPC among women who inject drugs [202]. Women reported that one of their reasons for using Insite was the protection it offered from having drugs taken from them by violence or intimidation [108]. Legal restrictions on peer injecting may have particular impacts on women, who are more likely to use this way [70,129,199,203]. Women may also be more exposed to injection-related injuries and disease [204].

The risk of male violence can be reduced and feelings of safety for women can be increased by providing women-only services, staffed by women-only teams (as at the Ragazza OPC in Hamburg), or extended opening hours when other spaces of safety are not available [23,70,114]. Such risks can also be mitigated by creating an environment that is more welcoming for women (e.g. a staff group that is largely made up of women with lived experience of street-based drug use). The evaluation that reported that it provided this woman-friendly environment was of the only mixed-gender OPC we found to report that a majority of its users were women [75].

People of minoritised ethnicities – including Indigenous people – who use drugs have high rates of overdose death and are more vulnerable to adverse policing while on the street [113,115]. Their experiences of racist discrimination or the provision of cultural safety will impact their use of OPCs. Use of the Melbourne’s medically supervised injecting room was frequent among Aboriginal and Torres Strait Islander people, who represented over 10% of this OPC’s users [19,163]. Racially minoritised people have reported reluctance to use OPCs, due to discrimination and stigma, in two studies [90,205], although another study found higher willingness to use an OPC in racially minoritised people who inject drugs [202]. Some OPCs have taken deliberate steps to support racially minoritised people. For example, providing space for Indigenous practices, recruiting an ethnically diverse staff group and providing training in cultural safety [114,125,206].

The combinations of contexts, mechanisms and outcomes that we found in reviewed documents are highly intersectional. For example, the observed reductions in deaths near Vancouver’s first OPC were higher among both women and people of First Nations ancestry [142], although Indigenous people who used Insite were less likely to enter addiction treatment [184]. This highlights the need for a gender-sensitive and culturally appropriate approach for creating safety, trust and social inclusion.

## 4 Discussion

This article presents the main causal pathway that we identified from our thematic, abductive and retroductive analysis of 391 selected documents. As the vast majority of the literature comes for OPCs in highly developed countries, our findings may only be relevant to OPCs in such settings. Our realist review adds to knowledge about the underlying generative mechanisms by which OPCs are expected and observed to produce their effects. By focusing on the mechanisms of safety, trust and social inclusion - and on how they interact with the preexisting and dynamic contexts of OPCs - policy makers and operators of OPCs can hope to maximise the benefits of providing these potentially life-saving and enhancing services.

Whereas most previous reviews of OPCs have focused on these services as discrete interventions that do or do not have effects, we found a more complex reality in which the outcomes of OPCs are contingent on specific combinations of contexts and mechanisms. The broader range of evidence included in our review enabled us to examine how OPCs operate in contexts characterised by violence, vulnerability and exclusion, and to collate evidence on the traces that the underlying causal mechanisms of OPCs produce in observable outcomes. A particular strength of this review, compared to others, is the inclusion of grey literature that is not archived in bibliographic databases. This enabled us to report, for example, on the few deaths that have occurred in OPCs, more information on the OPCs in Europe, and the existence of an OPC of which the majority of users are women [61,75].

In common with many existing studies of OPCs, we cannot provide definitive conclusions on their causal effects. The practical difficulty of running experimental trials of OPCs mean that it is unlikely that studies will meet the threshold for high quality causal evidence that is used in some systematic reviews [207]. Although one such review of OPC outcomes reported the reviewed evidence to be of ‘good methodological quality’ [25], another rated the certainty of evidence as low or very low [27]. Other authors have raised doubts about the rigour of the evidence base for OPCs [208].

In this new review, we do not seek to provide a definitive test of whether OPCs generally ‘work’ in producing posited benefits. To do so would clash with our critical realist assumption that the effects of interventions do not follow universally applicable laws but rather depend on specific, contingent combinations of contexts and mechanisms [209]. In realist thinking, decontextualised experiments are not sufficient alone to inform the implementation of complex interventions like OPCs [210].

Future research in this area can use the theorised causal pathway that is presented here to inform their questions and designs. We are already using the findings of this review in the development of a Core Outcome Set for OPCs [211]. We intend to carry out more research which uses quasi-experimental comparisons, administrative data linkage and health surveillance in between-site comparison to test the causal effect, learning from the existing studies that have informed us of these outcomes. We invite other researchers to also use review findings in this collaborative effort.

OPCs are not the only interventions that link people who use drugs to services that can improve their health and living conditions. This makes it difficult to disentangle the effects of OPCs from other harm reduction, treatment and social services. Our review suggests that in many of the places that OPCs have been established, their users find that the OPC plays a crucial role – which has not been fully played by these other services – in providing spaces of safety, trust and social inclusion.

## 5 Conclusions

The causal pathway we present here from our realist review can be summarised as follows. If OPCs succeed in providing an experience of safety for people who are otherwise exposed to high levels of drug-related risk and other forms of harm and violence, then they can build the necessary trust to support trajectories towards social inclusion and improved health, because providing safety both reduces the risk of dying and becoming infected, but also creates a platform of trust from which people can build connections to people and services that can help them overcome the various adversities they face.

## Supplementary Material

Additional supporting information can be found online in the Supporting Information section at the end of this article.

Supplementary Material

## Figures and Tables

**Table 1:** Details of literature search for the realist review on overdose prevention centres.

Dates of search	18-20 April 2023
Databases and hits	SCOPUS – 1008 Pubmed – 664 Web of Science – 986 ISSDP – 10
Search terms	“overdose prevention cent*” OR “overdose prevention site*” OR “overdose prevention programme*” OR “overdose prevention facilit*” OR “supervised inject* service*” OR “supervised inject* facilit*” OR “supervised inject* centre*” OR “supervised inject*” OR “supervised inject* programme*” OR “supervised inject* room*” OR “supervised fixing room*” OR “supervised drug consumption facilit*” OR “supervised injectable maintenance clinic*” OR “safe* inject* facilit*” OR “safe* inject* space*” OR “safe* consumption space*” OR “drug consumption room*” OR “drug consumption facilit*” OR “medically supervised inject* cent*” OR “fix* room*” OR “safe* environment intervention*” OR “shooting galler*”
Inclusion criteria	Providing empirical data on actually existing overdose prevention centres Written in English
Exclusion criteria	Written in another language than English Feasibility studies Opinion pieces Commentaries Policy reports

**Table 2:** Research methods used in the selected document.

Survey study	97	Time series analysis	7
Qualitative interview study	93	Scoping review	5
Cohort study	79	Pilot study	4
Narrative review	51	Chemical analysis	3
Ethnography	35	Choice experiment	3
Case study	25	Process evaluation	3
Systematic review	24	Participatory photography	3
Modelling study	22	Economic evaluation	2
Monitoring study	20	Legal analysis	2
Quasi-experimental evaluation	15	Ethical issues	1
Document analysis	14	Realist review	1
Policy analysis	10	Spatial mapping	1
Health surveillance	7	Randomised controlled trial	0

**Table 3:** Number of overdose prevention centres covered by selected documents by country.

Canada	30
Germany	30
The Netherlands	6
Australia	3
Denmark	3
Spain	3
United States	3
France	2
Belgium	1
Greece	1
Italy	1
Luxembourg	1
Mexico	1
Norway	1
Portugal	1
Switzerland	1
United Kingdom	1

## Footnotes

1 We recognise that there may be a preference for different terms and that terminology holds legal implications for their operation (e.g. in Canada), but for consistency we use OPC throughout this article.

2 In realist analysis, a program theory is ‘an abstracted description and/or diagram that lays out what a program (or family of programs or intervention) comprises and how it is expected to work’. An initial program theory is developed in order to sensitise researchers to contexts, mechanisms and outcomes that they may find in reviewed documents [38].

3 Since we completed our document search, a new study from Toronto has been published which also shows reductions in overdose deaths in the vicinity of nine OPCs [212].
