# Supplementary material for: Overdose prevention centres as spaces of safety, trust and inclusion: A causal pathway based on a realist review
Source: Drug Alcohol Rev. Author manuscript; Available in PMC 2025 Jul 29. (PMC7617959; doi:10.1111/dar.13908)
Supplement: Supplementary Material [file EMS206922-supplement-Supplementary_Material.zip › dar13908-sup-0003-appendix3.docx]

**Appendix 3: List of overdose prevention centres mentioned in selected documents**

| **Country** | **City** | **OPC** |
| --- | --- | --- |
| Australia | Melbourne | Melbourne MSIR |
|  | Sydney | Sydney Tolerance Room |
|  |  | The Sydney MSIC |
| Belgium | Liege | Liege DCR |
| Canada |  | Western Canada hospital-based SCS |
|  | Calgary | Beltline SCS |
|  |  | Safeworks |
|  | Kamloops | Kamloops mobile OPC |
|  | Kelowna | Kelowna mobile OPC |
|  | Lethbridge | Lethbridge OPC |
|  |  | Lethbridge SCS |
|  | London | Carepoint Consumption & Treatment Service |
|  | Ottawa | The Trailer |
|  | Surrey | SCS Safepoint Surrey |
|  | Toronto | Casey House SCS |
|  |  | Moss Park SCS |
|  |  | Queen West Community Health Centre |
|  |  | St Stephen’s OPS |
|  |  | Street Health OPS |
|  |  | The Works |
|  |  | Regent Park |
|  |  | South Riverdale |
|  |  | Toronto Unsanctioned OPC |
|  | Vancouver | 327 Carrel Street |
|  |  | Dr Peter Centre |
|  |  | Insite |
|  |  | Molson OPS and Learning Lab |
|  |  | Pop Up SIS |
|  |  | SisterSpace Vancouver |
|  |  | Thomus Donaghy OPC St. Paul's Hospital |
|  |  | VANDU’s Unsanctioned Inhalation Facility |
|  |  | VANDU’s unsanctioned SIF |
| Denmark | Aarhus | Aarhus DCR |
|  | Copenhagen | Maendenes Hjem |
|  |  | Skyen |
| France | Paris |  |
|  | Strasbourg |  |
| Germany | Aachen | Drogenhilfe Aachen |
|  |  | Suchthilfe Aachen |
|  | Berlin | Birkenstube |
|  |  | Fixpunkt |
|  |  | SKA |
|  | Bielefield | Bielefield DCR |
|  |  | Drogenberatung Bielefeld e.V. |
|  | Bochum | Krisenhilfe Bochum |
|  | Bonn | DCR Bonn |
|  |  | Verein fur Gefahrdetenhilfe |
|  | Dortmund | KICK – Aide-Hilfe |
|  | Dusseldorf | Dusseldorger Drogenhilfe e.V. |
|  | Essen | The Essen DCR |
|  | Frankfurt | Drogennotdienst Frankfurt |
|  |  | Eastside |
|  |  | Elbestraße |
|  |  | La Strada |
|  |  | Nidda 49 |
|  | Hamburg | Drob Inn |
|  |  | DroBill |
|  |  | Fixstern |
|  |  | Kodrobs Altona |
|  |  | Ragazza e.V. |
|  |  | Stay Alive |
|  | Koln | KAD I |
|  |  | KAD II |
|  |  | Kontakstelle fur Drogenabhangige |
|  | Munster | Indro |
|  | Saarbrucken | Drogenhilfezentrum Saarbrucken gGmbH |
|  | Wuppertal | Gleis 1 |
| Greece | Athens | DCR Athens |
| Luxembourg | Abrigado | Abrigado DCR |
| Mexico | Mexicali | Verter SCS |
| Netherlands | Amsterdam | AMOC |
|  |  | Stichting Gelders Centrum Voor Verslavingszorg |
|  | Rotterdam | Buurthuis |
|  |  | Keetje Tippel |
|  |  | Moerkerkestraat |
|  |  | Pauluskerk |
| Norway | Oslo | Oslo SCS |
| Portugal | Lisbon | Lisbon’s mobile DCR |
| Scotland | Glasgow | Glasgow’s unsanctioned OPC |
| Spain | Barcelona | CAS Baluard |
| Switzerland | Geneva | Quai 9 |
| USA |  | US unsanctioned SCS |
|  | New York City | Onpoint NYC |
|  |  | Portapotty SCS |

MSIR, Medically Supervised Injecting Room; OPC, overdose prevention centres; SCS, Safe Consumption Site
