# Supplementary material for: Overdose prevention centres as spaces of safety, trust and inclusion: A causal pathway based on a realist review
Source: Drug Alcohol Rev. Author manuscript; Available in PMC 2025 Jul 29. (PMC7617959; doi:10.1111/dar.13908)
Supplement: Supplementary Material [file EMS206922-supplement-Supplementary_Material.zip › dar13908-sup-0002-appendix2.docx]

**Appendix 2: Full list of selected documents**

Alberta Community Council on HIV. (2019). A Community Based Report on Alberta’s Supervised Consumption Service Effectiveness. Available from: <https://crismprairies.ca/wp-content/uploads/2019/08/A-Community-Based-Report-on-Alberta%E2%80%99s-SCS-Effectiveness-2019-08-16.pdf>

Andresen, M. A., & Boyd, N. (2010). A cost-benefit and cost-effectiveness analysis of Vancouver’s supervised injection facility. *International Journal of Drug Policy*, *21*(1), 70–76. <https://doi.org/10.1016/j.drugpo.2009.03.004>

Andresen, M. A., & Jozaghi, E. (2012). The point of diminishing returns: an examination of expanding Vancouver’s Insite. *Urban Studies*, *49*(16), 3531–3544. https://doi.org/10.1177/0042098012443865

Ankjærgaard, S. K., Christensen, I., Ege, P. P., Gotfredsen, N. W., Kjær, J., Olsen, M. L., & Majlund, K. L. (2015). From civil disobedience to drug users’ well-being: grass-roots activity and the establishment of drug consumption rooms in Denmark. *Drugs and Alcohol Today*, *15*(3), 141–148. https://doi.org/10.1108/DAT-03-2015-0007

Anoro, M., Ilundain, E., & Santisteban, O. (2003). Barcelona’s safer injection facility - EVA: A harm reduction program lacking official support. *Journal of Drug Issues*, *33*(3), 689–711. https://doi.org/10.1177/002204260303300309

Armbrecht, E., Guzauskas, G., Hansen, R., Pandey, R., Fazioli, K., Chapman, R., Pearson, S. D., & Rind, D. M. (2021). Supervised Injection Facilities and Other Supervised Consumption Sites: Effectiveness and Value. Final Report. Available from <https://icer.org/wp-content/uploads/2020/10/ICER_SIF_Final-Evidence-Report_010821.pdf>

Arredondo-Sánchez Lira, J., Fleiz-Bautista, C., Baker, P., Villatoro-Velázquez, J. A., Domínguez-García, M., & Beletsky, L. (2019). Attitudes towards safe consumption sites among police and people with lived experience in Tijuana, Mexico: initial report from the field. *Salud Mental*, *42*(4), 185–189. https://doi.org/10.17711/sm.0185-3325.2019.024

Atkinson, A. M., McAuley, A., Trayner, K. M. A., & Sumnall, H. R. (2019). ‘We are still obsessed by this idea of abstinence’: A critical analysis of UK news media representations of proposals to introduce drug consumption rooms in Glasgow, UK. *International Journal of Drug Policy*, *68*, 62–74. https://doi.org/10.1016/j.drugpo.2019.03.010

Axelsson, A., Søholm, H., Dalsgaard, M., Helweg-Larsen, J., Ihlemann, N., Bundgaard, H., Køber, L., & Iversen, K. (2014). Echocardiographic findings suggestive of infective endocarditis in asymptomatic Danish injection drug users attending urban injection facilities. *American Journal of Cardiology*, *114*(1), 100–104. https://doi.org/10.1016/j.amjcard.2014.04.010

Baker, P., Beletsky, L., Avalos, L., Venegas, C., Rivera, C., Strathdee, S. A., & Cepeda, J. (2020). Policing Practices and Risk of HIV Infection among People Who Inject Drugs. *Epidemiologic Reviews*, *42*(1), 27–40. https://doi.org/10.1093/epirev/mxaa010

Baker, T., & McCann, E. (2020). Beyond failure: The generative effects of unsuccessful proposals for Supervised Drug Consumption Sites (SCS) in Melbourne, Australia. *Urban Geography, 41,* 1179-1197. https://doi.org/10.1080/02723638.2018.1500254

Bardwell, G., Strike, C., Altenberg, J., Barnaby, L., & Kerr, T. (2019). Implementation contexts and the impact of policing on access to supervised consumption services in Toronto, Canada: A qualitative comparative analysis. *Harm Reduction Journal*, *16*(1). https://doi.org/10.1186/s12954-019-0302-x

Bardwell, G., Strike, C., Mitra, S., Scheim, A., Barnaby, L., Altenberg, J., & Kerr, T. (2020). “That’s a double-edged sword”: Exploring the integration of supervised consumption services within community health centres in Toronto, Canada. *Health and Place*, *61*, 102245. https://doi.org/10.1016/j.healthplace.2019.102245

Barratt, M. J., Latimer, J., Jauncey, M., Tay, E., & Nielsen, S. (2018). Urine drug screening for early detection of unwitting use of fentanyl and its analogues among people who inject heroin in Sydney, Australia. *Drug and Alcohol Review*, *37*(7), 847–850. https://doi.org/10.1111/dar.12864

Barry, C., Sherman, S., Stone, E., Kennedy-Hendricks, A., Niederdeppe, J., Linden, S., & McGinty, E. (2019). Arguments supporting and opposing legalization of safe consumption sites in the US. *International Journal of Drug Policy*, *63*, 18–22. https://doi.org/10.1016/j.drugpo.2018.10.008

Bayoumi, A. M., Strike, C., Brandeau, M., Degani, N., Fischer, B., Glazier, R., Hopkins, S., Leonard, L., Luce, J., Millson, P., O’Campo, P., Shepherd, S., Smith, C., & Zaric, G. S. (2012). Report of the Toronto and Ottawa supervised consumption assessment study, 2012. Available from: <https://www.catie.ca/sites/default/files/TOSCA%20report%202012.pdf>

Bayoumi, A. M., & Zaric, G. S. (2008). The cost-effectiveness of Vancouver’s supervised injection facility. *CMAJ. Canadian Medical Association Journal*, *179*(11), 1143–1151. https://doi.org/10.1503/cmaj.080808

Behrends, C. N., Paone, D., Nolan, M. L., Tuazon, E., Murphy, S. M., Kapadia, S. N., Jeng, P. J., Bayoumi, A. M., Kunins, H. V., & Schackman, B. R. (2019). Estimated impact of supervised injection facilities on overdose fatalities and healthcare costs in New York City. *Journal of Substance Abuse Treatment*, *106*, 79–88. https://doi.org/10.1016/j.jsat.2019.08.010

Belackova, V., & Salmon, A. (2017). Overview of International Literature – Supervised Injecting Facilities & Drug Consumption Rooms. Available from: https://www.drugsandalcohol.ie/34158/1/MISC_Supervised_injecting_overview_international_literature.pdf

Belackova, V., Salmon, A. M., Schatz, E., & Jauncey, M. (2017a). Online census of Drug Consumption Rooms (DCRs) as a setting to address HCV: current practice and future capacity.

Belackova, V., Salmon, A. M., Schatz, E., & Jauncey, M. (2018). Drug consumption rooms (DCRs) as a setting to address hepatitis C - findings from an international online survey. *Hepatology, Medicine and Policy*, *3*, 9. https://doi.org/10.1186/s41124-018-0035-6

Belackova, V., Salmon, A., Schatz, E., & Jauncey, M. (2017b). Drug consumption rooms as a space to increase hepatitis C virus treatment coverage: an international survey. *Drug and Alcohol, 36,* 8-9.

Belackova, V., Silins, E., Salmon, A. M., Jauncey, M., & Day, C. A. (2019). Beyond safer injecting—health and social needs and acceptance of support among clients of a supervised injecting facility. *International Journal of Environmental Research and Public Health*, *16*(11). https://doi.org/10.3390/ijerph16112032

Bell, S., & Globerman, J. (2014). Rapid Response Service. Effectiveness of supervised injection services. *Ontario HIV Treatment Network*, 1–8.

Bergamo, S., Parisi, G., & Jarre, P. (2019). Harm reduction in Italy: the experience of an unsanctioned supervised injection facility run by drug users. *Drugs and Alcohol Today*, *19*(2), 59–71. https://doi.org/10.1108/DAT-03-2018-0011

Berrigan, P., & Zucchelli, E. (2022). Public preferences for safe consumption sites for opioid use: A discrete choice experiment. *Drug and Alcohol Dependence*, *238*. https://doi.org/10.1016/j.drugalcdep.2022.109578

Betsos, A., Valleriani, J., Boyd, J., Bardwell, G., Kerr, T., & McNeil, R. (2021). “I couldn’t live with killing one of my friends or anybody”: A rapid ethnographic study of drug sellers’ use of drug checking. *International Journal of Drug Policy*, *87*. https://doi.org/10.1016/j.drugpo.2020.102845

Betsos, A., Valleriani, J., Boyd, J., & McNeil, R. (2022). Beyond co-production: The construction of drug checking knowledge in a Canadian supervised injection facility. *Social Science and Medicine*, *314*. https://doi.org/10.1016/j.socscimed.2022.115229

Bouchard, M., Hashimi, S., Tsai, K., Lampkin, H., & Jozaghi, E. (2018). Back to the core: A network approach to bolster harm reduction among persons who inject drugs. *International Journal of Drug Policy*, *51*, 95–104. https://doi.org/10.1016/j.drugpo.2017.10.006

Bourque, S., Pijl, E. M., Mason, E., Manning, J., & Motz, T. (2019). Supervised inhalation is an important part of supervised consumption services. *Canadian Journal of Public Health*, *110*(2), 210–215. https://doi.org/10.17269/s41997-019-00180-w

Bouzanis, K., Joshi, S., Lokker, C., Pavalagantharajah, S., Qiu, Y., Sidhu, H., Mbuagbaw, L., Qutob, M., Henedi, A., Levine, M. A. H., Lennox, R., Tarride, J.-E., Kalina, D., & Alvarez, E. (2021). Health programmes and services addressing the prevention and management of infectious diseases in people who inject drugs in Canada: A systematic integrative review. *BMJ Open*, *11*(9). https://doi.org/10.1136/bmjopen-2020-047511

Boyd, J., Collins, A. B., Mayer, S., Maher, L., Kerr, T., & McNeil, R. (2018). Gendered violence and overdose prevention sites: a rapid ethnographic study during an overdose epidemic in Vancouver, Canada. *Addiction*, *113*(12), 2261–2270. https://doi.org/10.1111/add.14417

Boyd, J., Lavalley, J., Czechaczek, S., Mayer, S., Kerr, T., Maher, L., & McNeil, R. (2020). “Bed Bugs and Beyond”: An ethnographic analysis of North America’s first women-only supervised drug consumption site. *International Journal of Drug Policy*, *78*. https://doi.org/10.1016/j.drugpo.2020.102733

Boyd, N. (2013). Lessons from INSITE, Vancouver’s supervised injection facility: 2003-2012. *Drugs: Education, Prevention and Policy*, *20*(3), 234–240. https://doi.org/10.3109/09687637.2012.755495

Bozinoff, N., Small, W., Long, C., DeBeck, K., & Fast, D. (2017). Still “at risk”: An examination of how street-involved young people understand, experience, and engage with “harm reduction” in Vancouver’s inner city. *International Journal of Drug Policy*, *45*, 33–39. https://doi.org/10.1016/j.drugpo.2017.05.006

Bravo, M. J., Royuela, L., De La Fuente, L., Brugal, M. T., Barrio, G., & Domingo-Salvany, A. (2009). Use of supervised injection facilities and injection risk behaviours among young drug injectors. *Addiction*, *104*(4), 614–619. https://doi.org/10.1111/j.1360-0443.2008.02474.x

Brinkley-Rubinstein, L., Cloud, D., Drucker, E., & Zaller, N. (2018). Opioid Use Among Those Who Have Criminal Justice Experience: Harm Reduction Strategies to Lessen HIV Risk. *Current HIV/AIDS Reports*, *15*(3), 255–258. https://doi.org/10.1007/s11904-018-0394-z

Broadhead, R. S., Kerr, T. H., Grund, J.-P. C., & Altice, F. L. (2002). Safer Injection Facilities in North America: Their Place in Public Policy and Health Initiatives. *Journal of Drug Issues*, *32*(1), 329–355. https://doi.org/10.1177/002204260203200113

Burris, S., Anderson, E. D., Beletsky, L., & David, C. S. (2009). Federalism, Policy Learning, and local innovation in Public Health: The case of the Supervised Injection Facility. *St. Louis University Law Journal*, *53*, 1089–1154. Available from: <https://scholarship.law.slu.edu/lj/vol53/iss4/7/>

Calgary Police, S. (2019). Crime & Disorder near the Sheldon M. Chumir Health Centre’s Supervised Consumption Services (SCS) Facility. Available from: <https://d3n8a8pro7vhmx.cloudfront.net/beltline/pages/388/attachments/original/1559446857/Q1_2019_CPS_Report.pdf?1559446857>

Cassie, R., Hayashi, K., DeBeck, K., Milloy, M. J., Cui, Z., Strike, C., West, J., & Kennedy, M. C. (2022). Difficulty accessing supervised consumption services during the COVID-19 pandemic among people who use drugs in Vancouver, Canada. *Harm Reduction Journal*, *19*(1). https://doi.org/10.1186/s12954-022-00712-7

Caulkins, J. P., Pardo, B., & Kilmer, B. (2019). Supervised consumption sites: a nuanced assessment of the causal evidence. *Addiction*, *114*(12), 2109–2115. https://doi.org/10.1111/add.14747

Centre for Organizational Effectiveness. (2018). Supervised Consumption Facilities: Community Consultation | London Ontario.

Cho, R., Purssell, R., Joe, R., Wang, Y. E., O’Sullivan, F., Lin, K., Rowe, A., & Moe, J. (2022). Opioid overdose and naloxone dosing at Insite supervised injection facility in British Columbia: A retrospective cohort study. *Canadian Journal of Addiction*, *13*(4), 22–31. https://doi.org/10.1097/CXA.0000000000000162

Cleirec, G., Fortias, M., Bloch, V., Clergue-Duval, V., Bellivier, F., Dusouchet, T., Debaulieu, C., & Vorspan, F. (2018). Opinion of health professionals and drug users before the forthcoming opening of the first drug consumption room in Paris: A quantitative cross-sectional study. *Harm Reduction Journal*, *15*(1). https://doi.org/10.1186/s12954-018-0260-8

Clua‐García, R., & Dumont, G. (2022). From the street to the drug consumption room. Injected drug use across consumption environments. *Ethnography*. https://doi.org/10.1177/14661381221113416

Collins, A. B., Boyd, J., Mayer, S., Fowler, A., Kennedy, M. C., Bluthenthal, R. N., Kerr, T., & McNeil, R. (2019). Policing space in the overdose crisis: A rapid ethnographic study of the impact of law enforcement practices on the effectiveness of overdose prevention sites. *The International Journal on Drug Policy*, *73*, 199–207. https://doi.org/10.1016/j.drugpo.2019.08.002

Collins, C. L. C., Kerr, T., Tyndall, M. W., Marsh, D. C., Kretz, P. S., Montaner, J. S., & Wood, E. (2005). Rationale to evaluate medically supervised safer smoking facilities for non-injection illicit drug users. *Canadian Journal of Public Health*, *96*(5), 344–347. https://doi.org/10.1007/bf03404029

Collins, C. L., Kerr, T., Kuyper, L. M., Li, K., Tyndall, M. W., Marsh, D. C., Montaner, J. S., & Wood, E. (2005). Potential uptake and correlates of willingness to use a supervised smoking facility for noninjection illicit drug use. *Journal of Urban Health*, *82*(2), 276–284. https://doi.org/10.1093/jurban/jti051

Constance, A. (2021). Safer Drug Consumption Facilites - Evidence Paper. Available from: <https://www.gov.scot/binaries/content/documents/govscot/publications/research-and-analysis/2021/10/safer-drug-consumption-facilites-evidence-paper/documents/safer-drug-consumption-facilities-evidence-paper/safer-drug-consumption-facilities-evidence-paper/govscot:document/safer-drug-consumption-facilities-evidence-paper.pdf>

Cortina, S., Kennedy, M. C., Dong, H., Fairbairn, N., Hayashi, K., Milloy, M. J., & Kerr, T. (2018). Willingness to use an in‐hospital supervised inhalation room among people who smoke crack cocaine in Vancouver, Canada. *Drug and Alcohol Review*, *37*(5), 645–652. https://doi.org/10.1111/dar.12815

Crescenzi, S. (2020). Measuring the social return on investment. A case study of Drug Consumption Rooms (DCRs) in Amsterdam. Available from: <https://smanjenje-stete.com/wp-content/uploads/2020/11/Measuring-the-SROI-thesis-final-version-Sofia-Crescenzi.pdf>

Cruz, M. F., Patra, J., Fischer, B., Rehm, J., & Kalousek, K. (2007). Public opinion towards supervised injection facilities and heroin-assisted treatment in Ontario, Canada. *International Journal of Drug Policy*, *18*(1), 54–61. https://doi.org/10.1016/j.drugpo.2006.12.001

Davidson, P. J., Lambdin, B. H., Browne, E. N., Wenger, L. D., & Kral, A. H. (2021). Impact of an unsanctioned safe consumption site on criminal activity, 2010–2019. *Drug and Alcohol Dependence*, *220*. https://doi.org/10.1016/j.drugalcdep.2021.108521

Davidson, P. J., Lopez, A. M., & Kral, A. H. (2018). Using drugs in un/safe spaces: Impact of perceived illegality on an underground supervised injecting facility in the United States. *International Journal of Drug Policy*, *53*, 37–44. https://doi.org/10.1016/j.drugpo.2017.12.005

Davidson, P. J., Wenger, L. D., Lambdin, B. H., & Kral, A. H. (2022). Establishment and enforcement of operational rules at an unsanctioned safe drug consumption site in the United States, 2014-2020. *Am J Public Health*, *112*, S166–S172. https://doi.org/10.2105/AJPH.2022.306714

Day, C. A., Salmon, A., Jauncey, M., Bartlett, M., & Roxburgh, A. (2022). Twenty-one years at the Uniting Medically Supervised Injecting Centre, Sydney: addressing the remaining questions. *Medical Journal of Australia*, *217*(8), 385–387. https://doi.org/10.5694/mja2.51716

De Gee, A., Woods, S., Charvet, C., & van der Poel, A. (2018). Drug Consumption Rooms in the Netherlands. Available from: <https://www.trimbos.nl/wp-content/uploads/sites/31/2021/09/af1684-drug-consumption-rooms-in-the-netherlands.pdf>

De Jong, W., & Weber, U. (1999). The professional acceptance of drug use: A closer look at drug consumption rooms in the Netherlands, Germany and Switzerland. *International Journal of Drug Policy*, *10*(2), 99–108. https://doi.org/10.1016/S0955-3959(98)00072-3

De Vel-Palumbo, M., Matthew-Simmons, F., Shanahan, M., & Ritter, A. (2013). Supervised Injecting Facilities: What the literature tells us. *Drug Policy Modelling Program*, *22*.

DeBeck, K., Kerr, T., Bird, L., Zhang, R., Marsh, D., Tyndall, M., Montaner, J., & Wood, E. (2011). Injection drug use cessation and use of North America’s first medically supervised safer injecting facility. *Drug and Alcohol Dependence*, *113*(2), 172–176. https://doi.org/10.1016/j.drugalcdep.2010.07.023

DeBeck, K., Kerr, T., Lai, C., Buxton, J., Montaner, J., & Wood, E. (2012). The validity of reporting willingness to use a supervised injecting facility on subsequent program use among people who use injection drugs. *American Journal of Drug and Alcohol Abuse*, *38*(1), 55–62. https://doi.org/10.3109/00952990.2011.600389

DeBeck, K., Wood, E., Zhang, R., Tyndall, M., Montaner, J., & Kerr, T. (2008). Police and public health partnerships: Evidence from the evaluation of Vancouver’s supervised injection facility. *Substance Abuse: Treatment, Prevention, and Policy*, *3*. https://doi.org/10.1186/1747-597X-3-11

Delvillano, S., de Groh, M., Morrison, H., & Do, M. T. (2019). Supervised injection services: A community-based response to the opioid crisis in the city of Ottawa, Canada. *Health Promotion and Chronic Disease Prevention in Canada*, *39*(3), 112–115. https://doi.org/10.24095/hpcdp.39.3.03

Dertadian, G. C., & Yates, K. (2023). “Overdose Has Many Faces”: The politics of care in responding to overdose at Sydney’s Medically Supervised Injecting Centre. *Contemporary Drug Problems*, *50*(1), 136–151. https://doi.org/10.1177/00914509221134716

Dertadian, G., & Tomsen, S. (2021). The experience of safety, harassment and social exclusion among male clients of Sydney’s Medically Supervised Injecting Centre. *International Journal for Crime, Justice and Social Democracy*, *10*(4). https://doi.org/10.5204/ijcjsd.2029

Des Jarlais, D. C., Arasteh, K., Semaan, S., & Wood, E. (2009). HIV among injecting drug users: Current epidemiology, biologic markers, respondent-driven sampling, and supervised-injection facilities. *Current Opinion in HIV and AIDS*, *4*(4), 308–313. https://doi.org/10.1097/COH.0b013e32832bbc6f

Dietze, P., Wilson, J., Whiteside, B., McLachlan, J., Vella-Horne, D., Clark, N., & Higgs, P. (2022). Changes in the use of Melbourne’s Medically Supervised Injecting Room (MSIR) over the COVID-19 pandemic. *IDRS.* https://doi.org/10.26190/drtz-3785

Diggles, J. H. (2020). Constitutional law - supervising consumption: The argument for supervised injection facilities as a valid exercise of states’ police power. *Western New England Law Review*, *42*(1), 95–127.

Dogherty, E., Patterson, C., Gagnon, M., Harrison, S., Chase, J., Boerstler, J., Gibson, J., Gill, S., Nolan, S., & Ryan, A. (2022). Implementation of a nurse-led overdose prevention site in a hospital setting: lessons learned from St. Paul’s Hospital, Vancouver, Canada. *Harm Reduction Journal*, *19*(1). <https://doi.org/10.1186/s12954-022-00596-7>

Dolan, K., Kimber, J., Fry, C., Fitzgerald, J., McDonald, D, & Trautmann, F. (2000). Drug consumption facilities in Europe and the establishment of supervised injecting centres in Australia. *Drug and Alcohol Review, 19*(3)*,* 337-326. http://doi.org/10.1080/713659379

Donnelly, N., & Mahoney, N. (2013). Trends in property and illicit drug crime around the Medically Supervised Injecting Centre in Kings Cross: 2012 update. Available from: <https://www.bocsar.nsw.gov.au/Publications/BB/bb90.pdf>

Dow-Fleisner, S. J., Lomness, A., & Woolgar, L. (2022). Impact of safe consumption facilities on individual and community outcomes: A scoping review of the past decade of research. *Emerging Trends in Drugs, Addictions, and Health*, *2*. https://doi.org/10.1016/j.etdah.2022.100046

Dubois-Arber, F., Balthasar, H., Huissoud, T., Zobel, F., Arnaud, S., Samitca, S., Jeannin, A., Schnoz, D., & Gervasoni, J. P. (2008). Trends in drug consumption and risk of transmission of HIV and hepatitis C virus among injecting drug users in Switzerland, 1993-2006. *Euro Surveillance : Bulletin Européen Sur Les Maladies Transmissibles, European Communicable Disease Bulletin*, *13*(21). https://doi.org/10.2807/ese.13.21.18881-en

Dubois-Arber, F., Benninghoff, F., & Jeannin, A. (2008). Typology of injection profiles of clients of a supervised drug consumption facility in Geneva, Switzerland. *European Addiction Research*, *14*(1), 1–10. https://doi.org/10.1159/000110405

Duncan, E., Shufelt, S., Barranco, M., & Udo, T. (2022). Acceptability of supervised injection facilities among persons who inject drugs in upstate New York. *Harm Reduction Journal*, *19*(1). https://doi.org/10.1186/s12954-022-00665-x

Duncan, T., Duff, C., Sebar, B., & Lee, J. (2017). ‘Enjoying the kick’: Locating pleasure within the drug consumption room. *International Journal of Drug Policy*, *49*, 92–101. https://doi.org/10.1016/j.drugpo.2017.07.005

Duncan, T., Sebar, B., Lee, J., & Duff, C. (2019). Mapping the spatial and affective composition of care in a drug consumption room in Germany. *Social and Cultural Geography*. https://doi.org/10.1080/14649365.2019.1610487

Duncan, T., Sebar, B., Lee, J., & Duff, C. (2020). Atmospheres of engagement within a German drug consumption room. *Social Science and Medicine*, *253*. https://doi.org/10.1016/j.socscimed.2020.112922

Dupree, T., Wood, C. I., & Brace, A. M. (2021). Understanding the stigma and feasibility of opening a safe injection facility in Baltimore city: A qualitative case study. *Qualitative Report*, *26*(6), 1911–1931. https://doi.org/10.46743/2160-3715/2021.4689

Elliott, D. (2014). Debating safe injecting sites in Vancouver’s inner city: Advocacy, conservatism and neoliberalism. *Contemporary Drug Problems*, *41*(1), 5–40. https://doi.org/10.1177/009145091404100102

European Monitoring Centre for Drugs and Drug Addiction. (2018). Drug consumption rooms: an overview of provision and evidence (Perspectives on drugs). Available from: <https://www.emcdda.europa.eu/publications/pods/drug-consumption-rooms_en>

Enns, E. A., Zaric, G. S., Strike, C. J., Jairam, J. A., Kolla, G., & Bayoumi, A. M. (2016). Potential cost-effectiveness of supervised injection facilities in Toronto and Ottawa, Canada. *Addiction*, *111*(3), 475–489. https://doi.org/10.1111/add.13195

Espelt, A., Villalbí, J. R., Bosque-Prous, M., Parés-Badell, O., Mari-Dell’Olmo, M., & Brugal, M. T. (2017). The impact of harm reduction programs and police interventions on the number of syringes collected from public spaces. A time series analysis in Barcelona, 2004–2014. *International Journal of Drug Policy*, *50*, 11–18. https://doi.org/10.1016/j.drugpo.2017.07.033

Fairbairn, N., Small, W., Shannon, K., Wood, E., & Kerr, T. (2008). Seeking refuge from violence in street-based drug scenes: Women’s experiences in North America’s first supervised injection facility. *Social Science and Medicine*, *67*(5), 817–823. https://doi.org/10.1016/j.socscimed.2008.05.012

Fairbairn, N., Small, W., Van Borek, N., Wood, E., & Kerr, T. (2010). Social structural factors that shape assisted injecting practices among injection drug users in Vancouver, Canada: A qualitative study. *Harm Reduction Journal*, *7*. https://doi.org/10.1186/1477-7517-7-20

Fast, D., Small, W., Wood, E., & Kerr, T. (2008). The perspectives of injection drug users regarding safer injecting education delivered through a supervised injecting facility. *Harm Reduction Journal*, *5*. https://doi.org/10.1186/1477-7517-5-32

Fernando, S., McNeil, R., Closson, K., Samji, H., Kirkland, S., Strike, C., Turje, R. B., Zhang, W., Hogg, R. S., & Parashar, S. (2016). An integrated approach to care attracts people living with HIV who use illicit drugs in an urban centre with a concentrated HIV epidemic. *Harm Reduction Journal*, *13*(1). https://doi.org/10.1186/s12954-016-0121-2

Fetene, M., Hall, C., & Dietze, P. (2020). Characteristics of people who used the Melbourne and Sydney medically supervised injecting facilities surveyed in the Illicit Drug Reporting System 2019. Available from: <https://www.drugsandalcohol.ie/33248/1/Melbourne-Sydney_MSIR_IDRS_Bulletin_final.pdf>

Fischer, B., Murphy, Y., Rudzinski, K., & MacPherson, D. (2016). Illicit drug use and harms, and related interventions and policy in Canada: A narrative review of select key indicators and developments since 2000. *International Journal of Drug Policy*, *27*, 23–35. https://doi.org/10.1016/j.drugpo.2015.08.007

Fitzgerald, J., Burgess, M., & Snowball, L. (2010). Trends in property and illicit drug crime around the Medically Supervised Injecting Centre in Kings Cross: An update. Available from: <https://www.bocsar.nsw.gov.au/Publications/BB/bb51.pdf>

Foglia, R., Kline, A., & Cooperman, N. A. (2021). New and Emerging Opioid Overdose Risk Factors. *Current Addiction Reports*, *8*(2), 319–329. https://doi.org/10.1007/s40429-021-00368-6

Folch, C., Lorente, N., Majó, X., Parés-Badell, O., Roca, X., Brugal, T., Roux, P., Carrieri, P., Colom, J., & Casabona, J. (2018). Drug consumption rooms in Catalonia: A comprehensive evaluation of social, health and harm reduction benefits. *International Journal of Drug Policy*, *62*, 24–29. https://doi.org/10.1016/j.drugpo.2018.09.008

Ford, R. (2010). An analysis of nurses’ views of harm reduction measures and other treatments for the problems associated with illicit drug use. *Australian Journal of Advanced Nursing*, *28*(1), 14–24.

Foreman-Mackey, A., Bayoumi, A. M., Miskovic, M., Kolla, G., & Strike, C. (2019). ‘It’s our safe sanctuary’: Experiences of using an unsanctioned overdose prevention site in Toronto, Ontario. *International Journal of Drug Policy*, *73*, 135–140. https://doi.org/10.1016/j.drugpo.2019.09.019

Foreman-Mackey, A., Kazatchkine, C., Elliott, R., & Ka Hon Chu, S. (2019). Overdue for a change: Scaling up Supervised Consumption Services in Canada. Available from: <https://www.hivlegalnetwork.ca/site/overdue-for-a-change-full-report/?lang=en>

Fortson, R. (2017). Setting Up a Drug Consumption Room Legal Issues. Available from: <https://www.rudifortson4law.co.uk/legaltexts/Rudi-Fortson-DCR-legal-issues-17thOct2017-v1.pdf>

Freeman, K., Jones, C. G. A., Weatherburn, D. J., Rutter, S., Spooner, C. J., & Donnelly, N. (2005). The impact of the Sydney Medically Supervised Injecting Centre (MSIC) on crime. *Drug and Alcohol Review*, *24*(2), 173–184. https://doi.org/10.1080/09595230500167460

Fry, C., Fox, S., & Rumbold, G. (1999). Establishing safe injecting rooms in Australia: attitudes of injecting drug users. *Australian and New Zealand Journal of Public Health*, *23*(5), 501–504. https://doi.org/10.1111/j.1467-842x.1999.tb01306.x

Fry, C. L. (2002). Injecting drug user attitudes towards rules for supervised injecting rooms: Implications for uptake. *International Journal of Drug Policy*, *13*(6), 471–476. https://doi.org/10.1016/S0955-3959(02)00076-2

Gaddis, A., Kennedy, M. C., Nosova, E., Milloy, M.-J., Hayashi, K., Wood, E., & Kerr, T. (2017). Use of on-site detoxification services co-located with a supervised injection facility. *Journal of Substance Abuse Treatment*, *82*, 1–6. https://doi.org/10.1016/j.jsat.2017.08.003

Gagnon, M., Gauthier, T., Adán, E., Bänninger, A., Cormier, L., Kathleen Gregg, J., Gill, S., Horsburgh, K., Kreutzmann, P., Latimer, J., Le Bourhis, G., Livgard, C., Parker, D., Reiremo, T., Telegdi, E., Thorner, T., & White, M. (2019). International Consensus Statement on the Role of Nurses in Supervised Consumption Sites. *The Journal of Mental Health and Addiction Nursing*, *3*(1), 22–31. https://doi.org/10.22374/jmhan.v3i1.35

Gehring, N. D., Speed, K. A., Launier, K., O’Brien, D., Campbell, S., & Hyshka, E. (2022). The state of science on including inhalation within supervised consumption services: A scoping review of academic and grey literature. *International Journal of Drug Policy*, *102*. https://doi.org/10.1016/j.drugpo.2022.103589

Gervasoni, J., Balthasar, H., Huissoud, T., Jeannin, A., & Dubois-Arber, F. (2012). A high proportion of users of low-threshold facilities with needle exchange programmes in Switzerland are currently on methadone treatment: Implications for new approaches in harm reduction and care. *International Journal of Drug Policy*, *23*(1), 33–36. https://doi.org/10.1016/j.drugpo.2011.05.015

Giglio, R. E., Mantha, S., Harocopos, A., Saha, N., Reilly, J., Cipriano, C., Kennelly, M., Landau, L., McRae, M., & Chokshi, D. A. (2023). The nation’s first publicly recognized overdose prevention centers: Lessons learned in New York City. *Journal of Urban Health*. https://doi.org/10.1007/s11524-023-00717-y

Gillies, M., Palmateer, N., Hutchinson, S., Ahmed, S., Taylor, A., & Goldberg, D. (2010). The provision of non-needle/syringe drug injecting paraphernalia in the primary prevention of HCV among IDU: A systematic review. *BMC Public Health*, *10*. https://doi.org/10.1186/1471-2458-10-721

Goodhew, M., Salmon, A. M., Marel, C., Mills, K. L., & Jauncey, M. (2016). Mental health among clients of the Sydney Medically Supervised Injecting Centre (MSIC). *Harm Reduction Journal*, *13*(1). https://doi.org/10.1186/s12954-016-0117-y

Goodman-Meza, D., Arredondo, J., Slim, S., Angulo, L., Gonzalez-Nieto, P., Loera, A., Shoptaw, S., Cambou, M. C., & Pitpitan, E. V. (2022). Behavior change after fentanyl testing at a safe consumption space for women in Northern Mexico: A pilot study. *International Journal of Drug Policy*, *106*. https://doi.org/10.1016/j.drugpo.2022.103745

Green, T. C., Hankins, C. A., Palmer, D., Boivin, J.-F., & Platt, R. (2004). My place, your place, or a safer place: The intention among Montréal injecting drug users to use supervised injecting facilities. *Canadian Journal of Public Health*, *95*(2), 110–114. https://doi.org/10.1007/bf03405777

Green, T., Hankins, C., Palmer, D., Boivin, J. F., & Platt, R. (2003). Ascertaining the need for a supervised injecting facility (SIF): The burden of public injecting in Montreal, Canada. *Journal of Drug Issues*, *33*(3), 713–731. https://doi.org/10.1177/002204260303300310

Greene, C., Maier, K., & Urbanik, M. M. (2023). “It’s just not the same”: Exploring PWUD’ perceptions of and experiences with drug policy and SCS services change in a Canadian City. *International Journal of Drug Policy*, *111*. https://doi.org/10.1016/j.drugpo.2022.103934

Greene, C., Urbanik, M.-M., & Geldart, R. (2022). Experiences with compounding surveillance and social control as a barrier to safe consumption service access. *SSM-Qualitative Research in Health*, *2.* https://doi.org/10.1016/j.ssmqr.2022.100055

Greenwald, Z. R., Bouck, Z., McLean, E., Mason, K., Lettner, B., Broad, J., Dodd, Z., Nassau, T., Scheim, A. I., & Werb, D. (2023). Integrated supervised consumption services and hepatitis C testing and treatment among people who inject drugs in Toronto, Canada: A cross-sectional analysis. *Journal of Viral Hepatitis*, *30*(2), 160–171. https://doi.org/10.1111/jvh.13780

Gubskaya, E., Kennedy, M. C., Hayashi, K., Cui, Z., Milloy, M.-J., & Kerr, T. (2023). The impact of the COVID-19 pandemic on access to supervised consumption programs. *Substance Abuse: Treatment, Prevention, and Policy*, *18*(1). https://doi.org/10.1186/s13011-023-00521-6

Hadland, S. E., Debeck, K., Kerr, T., Nguyen, P., Simo, A., Montaner, J. S., & Wood, E. (2014). Use of a medically supervised injection facility among street youth. *Journal of Adolescent Health*, *55*(5), 684–689. https://doi.org/10.1016/j.jadohealth.2014.04.013

Harocopos, A., Gibson, B. E., Saha, N., McRae, M. T., See, K., Rivera, S., & Chokshi, D. A. (2022). First 2 Months of Operation at First Publicly Recognized Overdose Prevention Centers in US. *JAMA Network Open*, *5*(7), E2222149. https://doi.org/10.1001/jamanetworkopen.2022.22149

Harris, R. E., Richardson, J., Frasso, R., & Anderson, E. D. (2018). Perceptions about supervised injection facilities among people who inject drugs in Philadelphia. *International Journal of Drug Policy*, *52*, 56–61. https://doi.org/10.1016/j.drugpo.2017.11.005

Havinga, P., Van der Velden, C., De Gee, A., & Van der Poel, A. (2014). Differences in sociodemographic, drug use and health characteristics between never, former and current injecting, problematic hard-drug users in the Netherlands. *Harm Reduction Journal*, *11*(1). https://doi.org/10.1186/1477-7517-11-6

Hayashi, K., Wood, E., Dong, H., Buxton, J. A., Fairbairn, N., DeBeck, K., Milloy, M.-J., & Kerr, T. (2021). Awareness of fentanyl exposure and the associated overdose risks among people who inject drugs in a Canadian setting. *Drug and Alcohol Review*, *40*(6), 964–973. https://doi.org/10.1111/dar.13261

Hedrich, D. (2004). European report on drug consumption rooms. Available from: <https://www.emcdda.europa.eu/html.cfm/index54125EN.html_en>

Houborg, E., & Frank, V. A. (2014). Drug consumption rooms and the role of politics and governance in policy processes. *International Journal of Drug Policy*, *25*(5), 972–977. https://doi.org/10.1016/j.drugpo.2014.01.008

Houborg, E., & Jauffret-Roustide, M. (2022). Drug consumption rooms: Welfare state and diversity in social acceptance in Denmark and in France. *American Journal of Public Health*, *112*, S159–S165. https://doi.org/10.2105/AJPH.2022.306808

Hunt, N., Lloyd, C., Kimber, J., & Tompkins, C. (2007). Public injecting and willingness to use a drug consumption room among needle exchange programme attendees in the UK. *International Journal of Drug Policy*, *18*(1), 62–65. https://doi.org/10.1016/j.drugpo.2006.11.018

Independent Working Group (2006). Drug consumption rooms: Summary report of the Independent Working Group. Available from: <https://www.jrf.org.uk/report/drug-consumption-rooms-summary-report-independent-working-group>

Irvine, M. A., Kuo, M., Buxton, J. A., Balshaw, R., Otterstatter, M., Macdougall, L., Milloy, M.-J., Bharmal, A., Henry, B., Tyndall, M., Coombs, D., & Gilbert, M. (2019). Modelling the combined impact of interventions in averting deaths during a synthetic-opioid overdose epidemic. *Addiction*, *114*(9), 1602–1613. https://doi.org/10.1111/add.14664

Irwin, A., Jozaghi, E., Bluthenthal, R. N., & Kral, A. H. (2017). A cost-benefit analysis of a potential supervised injection facility in San Francisco, California, USA. *Journal of Drug Issues*, *47*(2), 164–184. https://doi.org/10.1177/0022042616679829

Irwin, A., Jozaghi, E., Weir, B. W., Allen, S. T., Lindsay, A., & Sherman, S. G. (2017). Mitigating the heroin crisis in Baltimore, MD, USA: A cost-benefit analysis of a hypothetical supervised injection facility. *Harm Reduction Journal*, *14*(1). https://doi.org/10.1186/s12954-017-0153-2

Ivsins, A., Boyd, J., Mayer, S., Collins, A., Sutherland, C., Kerr, T., & McNeil, R. (2020). Barriers and facilitators to a novel low-barrier hydromorphone distribution program in Vancouver, Canada: a qualitative study. *Drug and Alcohol Dependence*, *216*. https://doi.org/10.1016/j.drugalcdep.2020.108202

Ivsins, A., Vancouver Area Network Of Drug Users, Benoit, C., Kobayashi, K., & Boyd, S. (2019). From risky places to safe spaces: Re-assembling spaces and places in Vancouver’s Downtown Eastside. *Health and Place*, *59.* https://doi.org/10.1016/j.healthplace.2019.102164

Ivsins, A., Warnock, A., Small, W., Strike, C., Kerr, T., & Bardwell, G. (2023). A scoping review of qualitative research on barriers and facilitators to the use of supervised consumption services. *International Journal of Drug Policy*, *111*. https://doi.org/10.1016/j.drugpo.2022.103910

Jackson, J. (2020). A cost analysis of overdose management at a supervised consumption site in Calgary, Canada. *Qeios*. https://doi.org/10.32388/j6mq0e

Jauffret-Roustide, M., & Cailbault, I. (2018). Drug consumption rooms: Comparing times, spaces and actors in issues of social acceptability in French public debate. *International Journal of Drug Policy*, *56*, 208–217. https://doi.org/10.1016/j.drugpo.2018.04.014

Jauncey, M., Livingston, M., Salmon, A. M., & Dietze, P. (2018). The impact of OxyContin reformulation at the Sydney Medically Supervised Injecting Centre: Pros and cons. *International Journal of Drug Policy*, *53*, 17–22. https://doi.org/10.1016/j.drugpo.2017.11.025

Javadi, R., Lagana, K., Krowicki, T., Bennett, D., & Schindler, B. (2022). Attitudes toward harm reduction among substance use treatment professionals in Philadelphia. *Journal of Substance Use*, *27*(5), 459–464. https://doi.org/10.1080/14659891.2021.1961320

Jordens, J., & Higgs, P. (2005). Couches vs Karaoke: Ethnic Vietnamese user views on safe injecting facilities in Melbourne, Australia. *New Community Quarterly*, *3*(1), 19–23.

Jozaghi, E. (2014). A cost-benefit/cost-effectiveness analysis of an unsanctioned supervised smoking facility in the Downtown Eastside of Vancouver, Canada.

Jozaghi, E. (2012). A little heaven in hell: The role of a supervised injection facility in transforming place. *Urban Geography*, *33*(8), 1144–1162. https://doi.org/10.2747/0272-3638.33.8.1144

Jozaghi, E., & Andresen, M. M. A. (2013). Should North America’s first and only supervised injection facility (InSite) be expanded in British Columbia, Canada? *Harm Reduction Journal*, *10*(1). https://doi.org/10.1186/1477-7517-10-1

Jozaghi, E., Hodgkinson, T., & Andresen, M. A. (2015). Is there a role for potential supervised injection facilities in Victoria, British Columbia, Canada? *Urban Geography*, *36*(8), 1241–1255. https://doi.org/10.1080/02723638.2015.1053200

Jozaghi, E., & Jackson, A. (2015). Examining the potential role of a supervised injection facility in Saskatoon, Saskatchewan, to avert HIV among people who inject drugs. *International Journal of Health Policy and Management*, *4*(6), 373–379. https://doi.org/10.15171/ijhpm.2015.73

Jozaghi, E., Lampkin, H., & Andresen, M. (2016). Peer-engagement and its role in reducing the risky behavior among crack and methamphetamine smokers of the Downtown Eastside community of Vancouver, Canada. *Harm Reduction Journal*, *13*. https://doi.org/10.1186/s12954-016-0108-z

Jozaghi, E., & Reid, A. A. (2014). A case study of the transformative effect of peer injection drug users in the Downtown Eastside of Vancouver, Canada. *Canadian Journal of Criminology and Criminal Justice*, *56*(5), 563–593. https://doi.org/10.1353/ccj.2014.0036

Jozaghi, E., & Reid, A. A. (2015). The potential role for supervised injection facilities in Canada’s largest city, Toronto. *International Criminal Justice Review*, *25*(3), 233–246. https://doi.org/10.1177/1057567715583516

Jozaghi, E., Reid, A. A., & Andresen, M. A. (2013). A cost-benefit/cost-effectiveness analysis of proposed supervised injection facilities in Montreal, Canada. *Substance Abuse: Treatment, Prevention, and Policy*, *8*(1). https://doi.org/10.1186/1747-597X-8-25

Jozaghi, E., Reid, A. A., Andresen, M. A., & Juneau, A. (2014). A cost-benefit/cost-effectiveness analysis of proposed supervised injection facilities in Ottawa, Canada. *Substance Abuse: Treatment, Prevention, and Policy*, *9*(1). https://doi.org/10.1186/1747-597X-9-31

Kappel, N., Toth, E., Tegner, J., & Lauridsen, S. (2016). A qualitative study of how Danish drug consumption rooms influence health and well-being among people who use drugs. *Harm Reduction Journal*, *13*(1). https://doi.org/10.1186/s12954-016-0109-y

Karamouzian, M., Dohoo, C., Forsting, S., McNeil, R., Kerr, T., & Lysyshyn, M. (2018). Evaluation of a fentanyl drug checking service for clients of a supervised injection facility, Vancouver, Canada. *Harm Reduction Journal*, *15*(1), 46. https://doi.org/10.1186/s12954-018-0252-8

Katz, N., Leonard, L., Wiesenfeld, L., Perry, J. J., Thiruganasambandamoorthy, V., & Calder, L. (2017). Support of supervised injection facilities by emergency physicians in Canada. *International Journal of Drug Policy*, *49*, 26–31. https://doi.org/10.1016/j.drugpo.2017.07.013

Kennedy, M. C., Boyd, J., Mayer, S., Collins, A., Kerr, T., & McNeil, R. (2019). Peer worker involvement in low-threshold supervised consumption facilities in the context of an overdose epidemic in Vancouver, Canada. *Social Science and Medicine*, *225*, 60–68. https://doi.org/10.1016/j.socscimed.2019.02.014

Kennedy, M. C., Hayashi, K., Milloy, M.-J., Boyd, J., Wood, E., & Kerr, T. (2020). Supervised injection facility use and exposure to violence among a cohort of people who inject drugs: A gender-based analysis. *International Journal of Drug Policy*, *78*. https://doi.org/10.1016/j.drugpo.2020.102692

Kennedy, M. C., Hayashi, K., Milloy, M.-J., Compton, M., & Kerr, T. (2022). Health impacts of a scale-up of supervised injection services in a Canadian setting: an interrupted time series analysis. *Addiction*, *117*(4), 986–997. https://doi.org/10.1111/add.15717

Kennedy, M. C., Hayashi, K., Milloy, M.-J., Wood, W., & Kerr, T. (2019). Supervised injection facility use and all-cause mortality among people who inject drugs in Vancouver, Canada: A cohort study. *PLoS Medicine*, *16*(11). https://doi.org/10.1371/journal.pmed.1002964

Kennedy, M. C., Karamouzian, M., & Kerr, T. (2017). Public health and public order outcomes associated with supervised drug consumption facilities: a systematic review. *Current HIV/AIDS Reports*, *14*(5), 161–183. https://doi.org/10.1007/s11904-017-0363-y

Kennedy, M. C., Klassen, D. C., Dong, H., Milloy, M.-J. S., Hayashi, K., & Kerr, T. H. (2019). Supervised injection facility utilization patterns: a prospective cohort study in Vancouver, Canada. *American Journal of Preventive Medicine*, *57*(3), 330–337. https://doi.org/10.1016/j.amepre.2019.04.024

Kennedy, M. C., Milloy, M.-J., Hayashi, K., Holliday, E., Wood, E., & Kerr, T. (2020). Assisted injection within supervised injection services: Uptake and client characteristics among people who require help injecting in a Canadian setting. *International Journal of Drug Policy*, *86*. https://doi.org/10.1016/j.drugpo.2020.102967

Kennedy, M. C., Scheim, A., Rachlis, B., Mitra, S., Bardwell, G., Rourke, S., & Kerr, T. (2018). Willingness to use drug checking within future supervised injection services among people who inject drugs in a mid-sized Canadian city. *Drug and Alcohol Dependence*, *185*, 248–252. https://doi.org/10.1016/j.drugalcdep.2017.12.026

Kennedy-Hendricks, A., Bluestein, J., Kral, A. H., Barry, C. L., & Sherman, S. G. (2019). Establishing sanctioned safe consumption sites in the United States: Five jurisdictions moving the policy agenda forward. *Psychiatric Services*, *70*(4), 294–301. https://doi.org/10.1176/appi.ps.201800398

Kenney, S. R., Anderson, B. J., Bailey, G. L., Herman, D. S., Conti, M. T., & Stein, M. D. (2021). Examining overdose and homelessness as predictors of willingness to use supervised injection facilities by services provided among persons who inject drugs. *American Journal on Addictions*, *30*(1), 21–25. https://doi.org/10.1111/ajad.13065

Kerman, N., Manoni-Millar, S., Cormier, L., Cahill, T., & Sylvestre, J. (2020). “It’s not just injecting drugs”: Supervised consumption sites and the social determinants of health. *Drug and Alcohol Dependence*, *213*, 108078. https://doi.org/10.1016/j.drugalcdep.2020.108078

Kerr, T., Oleson, M., Tyndall, M. W., Montaner, J., & Wood, E. (2005). A description of a peer-run supervised injection site for injection drug users. *Journal of Urban Health*, *82*(2), 267–275. https://doi.org/10.1093/jurban/jti050

Kerr, T., Small, W., Moore, D., & Wood, E. (2007). A micro-environmental intervention to reduce the harms associated with drug-related overdose: Evidence from the evaluation of Vancouver’s safer injection facility. *International Journal of Drug Policy*, *18*(1), 37–45. https://doi.org/10.1016/j.drugpo.2006.12.008

Kerr, T., Stoltz, J.-A., Tyndall, M., Li, K., Zhang, R., Montaner, J., & Wood, E. (2006). Impact of a medically supervised safer injection facility on community drug use patterns: A before and after study. *British Medical Journal*, *332*(7535), 220–222. https://doi.org/10.1136/bmj.332.7535.220

Kerr, T., Tyndall, M. W., Lai, C., Montaner, J. S. G., & Wood, E. (2006). Drug-related overdoses within a medically supervised safer injection facility. *International Journal of Drug Policy*, *17*(5), 436–441. https://doi.org/10.1016/j.drugpo.2006.05.008

Kerr, T., Tyndall, M. W., Zhang, R., Lai, C., Montaner, J. S. G., & Wood, E. (2007). Circumstances of first injection among illicit drug users accessing a medically supervised safer injection facility. *American Journal of Public Health*, *97*(7), 1228–1230. https://doi.org/10.2105/AJPH.2006.086256

Kerr, T., Wood, E., Palepu, A., Wilson, D., Schechter, M. T., & Tyndall, M. W. (2003). Responding to an explosive HIV epidemic driven by frequent cocaine injection: Is there a role for safe injecting facilities? *Journal of Drug Issues*, *33*(3), 579–608. https://doi.org/10.1177/002204260303300303

Kerr, T., Wood, E., Small, D., Palepu, A., & Tyndall, M. W. (2003). Potential use of safer injecting facilities among injection drug users in Vancouver’s Downtown Eastside. *CMAJ. Canadian Medical Association Journal*, *169*(8), 759–763. https://www.scopus.com/inward/record.uri?eid=2-s2.0-0142246431&partnerID=40&md5=1c46ae4779cc8926925b0dc363d7a79f

Kerr, T., Tyndall, M., Li, K., Montaner, J., & Wood, E. (2005). Safer injection facility use and syringe sharing in injection drug users. *Lancet*, *366*(9482), 316–318. https://doi.org/10.1016/S0140-6736(05)66475-6

Khair, S., Eastwood, C. A., Lu, M., & Jackson, J. (2022). Supervised consumption site enables cost savings by avoiding emergency services: a cost analysis study. *Harm Reduction Journal*, *19*(1), 1–7. https://doi.org/10.1186/s12954-022-00609-5

Kilmer, B., Taylor, J., Caulkins, J. P., Mueller, P. A., Ober, A. J., Pardo, B., Smart, R., Strang, L., & Reuter, P. H. (2018). Considering heroin-assisted treatment and supervised srug consumption sites in the United States. Available from: https://www.rand.org/pubs/research_reports/RR2693.html

Kimber, J., & Dolan, K. (2007). Shooting gallery operation in the context of establishing a medically supervised injecting center: Sydney, Australia. *Journal of Urban Health*, *84*(2), 255–266. https://doi.org/10.1007/s11524-006-9145-3

Kimber, J., Dolan, K., Van Beek, I., Hedrich, D., & Zurhold, H. (2003). Drug consumption facilities: An update since 2000. *Drug and Alcohol Review*, *22*(2), 227–233. https://doi.org/10.1080/095952301000116951

Kimber, J., Dolan, K., & Wodak, A. (2005). Survey of drug consumption rooms: Service delivery and perceived public health and amenity impact. *Drug and Alcohol Review*, *24*(1), 21–24. https://doi.org/10.1080/09595230500125047

Kimber, J., Hickman, M., Degenhardt, L., Coulson, T., & Van Beek, I. (2008). Estimating the size and dynamics of an injecting drug user population and implications for health service coverage: Comparison of indirect prevalence estimation methods. *Addiction*, *103*(10), 1604–1613. https://doi.org/10.1111/j.1360-0443.2008.02276.x

Kimber, J., Macdonald, M., Van Beek, I., Kaldor, J., Weatherburn, D., Lapsley, H., & Mattick, R. P. (2003). The Sydney Medically Supervised Injecting Centre: Client characteristics and predictors of frequent attendance during the first 12 months of operation. *Journal of Drug Issues*, *33*(3), 639–648. https://doi.org/10.1177/002204260303300306

Kimber, J., Mattick, R. P., Kaldor, J., Van Beek, I., Gilmour, S., & Rance, J. A. (2008). Process and predictors of drug treatment referral and referral uptake at the Sydney Medically Supervised Injecting Centre. *Drug and Alcohol Review*, *27*(6), 602–612. https://doi.org/10.1080/09595230801995668

Kinnard, E. N., Howe, C. J., Kerr, T., Hass, V. S., & Marshall, B. D. L. (2014). Self-reported changes in drug use behaviors and syringe disposal methods following the opening of a supervised injecting facility in Copenhagen, Denmark. *Harm Reduction Journal*, *11*(1). https://doi.org/10.1186/1477-7517-11-29

Kinshella, M.-L. W., Gauthier, T., & Lysyshyn, M. (2018). Rigidity, dyskinesia and other atypical overdose presentations observed at a supervised injection site, Vancouver, Canada. *Harm Reduction Journal*, *15*(1). https://doi.org/10.1186/s12954-018-0271-5

Klein, K. S., Glick, S. N., & Mauro, P. M. (2020). Anticipated use of a supervised drug consumption site among syringe services program clients in King County, Washington: Assessing the role of opioid overdose and injection behavior. *Drug and Alcohol Dependence*, *213*. https://doi.org/10.1016/j.drugalcdep.2020.108121

Knecht, R., Bemister-Williams, G., Els, C., Hollihan, J., Tanguay, R., Baker, R., Maxim, P., & Cormack, S. (2020). Impact: A socio-economic review of supervised consumption sites in Alberta. Available from: <https://open.alberta.ca/publications/9781460147054>

Kolla, G., Kenny, K. S., Bannerman, M., Boyce, N., Chapman, L., Dodd, Z., Ko, J., & Ovens, S. (2020). Help me fix: The provision of injection assistance at an unsanctioned overdose prevention site in Toronto, Canada. *International Journal of Drug Policy*, *76*. https://doi.org/10.1016/j.drugpo.2019.102617

Kolla, G., Penn, R., & Long, C. (2019). Evaluation of the Overdose Prevention Sites at Street Health and St. Stephen’s Community House. Available from: <https://streethealth.ca/wp-content/uploads/2023/01/ops-full-evaluation.pdf>

Kolla, G., Strike, C., Watson, T. M., Jairam, J., Fischer, B., & Bayoumi, A. M. (2017). Risk creating and risk reducing: Community perceptions of supervised consumption facilities for illicit drug use. *Health, Risk and Society*, *19*(1), 91–111. https://doi.org/10.1080/13698575.2017.1291918

Kosteniuk, B., Salvalaggio, G., McNeil, R., Brooks, H. L., Dong, K., Twan, S., Brouwer, J., & Hyshka, E. (2021). “You don’t have to squirrel away in a staircase”: Patient motivations for attending a novel supervised drug consumption service in acute care. *International Journal of Drug Policy*, *96*. https://doi.org/10.1016/j.drugpo.2021.103275

Kral, A. H., Lambdin, B. H., Wenger, L. D., Browne, E. N., Suen, L. W., & Davidson, P. J. (2021). Improved syringe disposal practices associated with unsanctioned safe consumption site use: A cohort study of people who inject drugs in the United States. *Drug and Alcohol Dependence*, *229*. https://doi.org/10.1016/j.drugalcdep.2021.109075

Kral, A. H., Wenger, L., Carpenter, L., Wood, E., Kerr, T., & Bourgois, P. (2010). Acceptability of a safer injection facility among injection drug users in San Francisco. *Drug and Alcohol Dependence*, *110*(1), 160–163. https://doi.org/10.1016/j.drugalcdep.2010.02.009

Krusi, A., Small, W., Wood, E., & Kerr, T. (2009). An integrated supervised injecting program within a care facility for HIV-positive individuals: A qualitative evaluation. *AIDS Care - Psychological and Socio-Medical Aspects of AIDS/HIV*, *21*(5), 638–644. https://doi.org/10.1080/09540120802385645

Kulesza, M., Teachman, B. A., Werntz, A. J., Gasser, M. L., & Lindgren, K. P. (2015). Correlates of public support toward federal funding for harm reduction strategies. *Substance Abuse: Treatment, Prevention, and Policy*, *10*(1). https://doi.org/10.1186/s13011-015-0022-5

Lafferty, L., Treloar, C., van Breda, N., Steele, M., Hiley, S., Flaherty, I., & Salmon, A. (2017). ‘It’s Fast, It’s Quick, It Stops Me Being Sick’: How to influence preparation of opioid tablets for injection. *Drug and Alcohol Review*, *36*(5), 651–657. https://doi.org/10.1111/dar.12562

Lalanne, L., Roux, P., Donadille, C., Briand Madrid, L., Celerier, I., Chauvin, C., Hamelin, N., Kervran, C., Maradan, G., Auriacombe, M., Jauffret-Roustide, M., & The COSINUS study group. (2023). Drug consumption rooms are effective to reduce at-risk practices associated with HIV/HCV infections among people who inject drugs: Results from the COSINUS cohort study. *Addition*. https://doi.org/10.1111/add.16320

Lam, T., Barratt, M. J., Bartlett, M., Latimer, J., Jauncey, M., Hiley, S., Clark, N., Gerostamoulos, D., Glowacki, L., Roux, C., Morelato, M., & Nielsen, S. (2022). Infrequent detection of unintentional fentanyl use via urinalysis among people who regularly inject opioids in Sydney and Melbourne, Australia. *Addiction*, *117*(8), 2331–2337. https://doi.org/10.1111/add.15832

Lange, B. C. L., & Bach-Mortensen, A. M. (2019). A systematic review of stakeholder perceptions of supervised injection facilities. *Drug and Alcohol Dependence*, *197*, 299–314. https://doi.org/10.1016/j.drugalcdep.2019.02.006

Larson, S., Padron, N., Mason, J., & Bogaczyk, T. (2017). Supervised Consumption Facilities - Review of the Evidence. Available from: <https://dbhids.org/wp-content/uploads/2018/01/OTF_LarsonS_PHLReportOnSCF_Dec2017.pdf>

Latimer, J., Ling, S., Flaherty, I., Jauncey, M., & Salmon, A. M. (2016). Risk of fentanyl overdose among clients of the Sydney Medically Supervised Injecting Centre. *International Journal of Drug Policy*, *37*, 111–114. https://doi.org/10.1016/j.drugpo.2016.08.004

Lefrancois, E., Belackova, V., Silins, E., Latimer, J., Jauncey, M., Shimmon, R., Mozaner Bordin, D., Augsburger, M., Esseiva, P., Roux, C., & Morelato, M. (2020). Substances injected at the Sydney supervised injecting facility: A chemical analysis of used injecting equipment and comparison with self-reported drug type. *Drug and Alcohol Dependence*, *209*. https://doi.org/10.1016/j.drugalcdep.2020.107909

León, C., Cardoso, L., Mackin, S., Bock, B., & Gaeta, J. M. (2018). The willingness of people who inject drugs in Boston to use a supervised injection facility. *Substance Abuse*, *39*(1), 95–101. https://doi.org/10.1080/08897077.2017.1365804

Levengood, T. W., Yoon, G. H., Davoust, M. J., Ogden, S. N., Marshall, B. D. L., Cahill, S. R., & Bazzi, A. R. (2021). Supervised injection facilities as harm reduction: A systematic review. *American Journal of Preventive Medicine*, *61*(5), 738–749. https://doi.org/10.1016/j.amepre.2021.04.017

Liang, J., & Alexeev, S. (2023). Harm reduction or amplification? The adverse impact of a supervised injection room on housing prices. *Regional Science and Urban Economics*, *98*. https://doi.org/10.1016/j.regsciurbeco.2022.103856

Lingle, C. (2013). A Critical Review of the effectiveness of Safe Injection Facilities as a Harm Reduction strategy. Available from: <https://d-scholarship.pitt.edu/18375/#:~:text=Safe%20injection%20facilities%20have%20an,of%20life%20for%20drug%20users>.

Lloyd, C., Stover, H., Zurhold, H., & Hunt, N. (2016). Similar problems, divergent responses: drug consumption room policies in the UK and Germany. *Journal of Substance Use, 22*(1), 66-70. https://doi.org/10.3109/14659891.2016.1143049

Lloyd-Smith, E., Hull, M. W., Tyndall, M. W., Zhang, R., Wood, E., Montaner, J. S. G., Kerr, T., & Romney, M. G. (2010). Community-associated methicillin-resistant Staphylococcus aureus is prevalent in wounds of community-based injection drug users. *Epidemiology and Infection*, *138*(5), 713–720. https://doi.org/10.1017/S0950268810000464

Lloyd-Smith, E., Tyndall, M., Zhang, R., Grafstein, E., Sheps, S., Wood, E., Montaner, J., & Kerr, T. (2012). Determinants of cutaneous injection-related infections among injection drug users at an emergency department. *Open Infectious Diseases Journal*, *6*(1), 5–11. https://doi.org/10.2174/1874279301206010005

Lloyd-Smith, E., Wood, E., Zhang, R., Tyndall, M. W., Montaner, J. S. G., & Kerr, T. (2008). Risk factors for developing a cutaneous injection-related infection among injection drug users: A cohort study. *BMC Public Health*, *8*. https://doi.org/10.1186/1471-2458-8-405

Lloyd-Smith, E., Wood, E., Zhang, R., Tyndall, M. W., Montaner, J. S., & Kerr, T. (2009). Determinants of cutaneous injection-related infection care at a supervised injecting facility. *Annals of Epidemiology*, *19*(6), 404–409. https://doi.org/10.1016/j.annepidem.2009.03.007

Lloyd-Smith, E., Wood, E., Zhang, R., Tyndall, M. W., Sheps, S., Montaner, J. S., & Kerr, T. (2010). Determinants of hospitalization for a cutaneous injection-related infection among injection drug users: A cohort study. *BMC Public Health*, *10*. https://doi.org/10.1186/1471-2458-10-327

Luchenski, S., Maguire, N., Aldridge, R. W., Hayward, A., Story, A., Perri, P., Withers, J., Clint, S., Fitzpatrick, S., & Hewett, N. (2018). What works in inclusion health: overview of effective interventions for marginalised and excluded populations. *The Lancet*, *391*, 266–280. https://doi.org/https://doi.org/10.1016/S0140-6736(17)31959-1

MacArthur, G. J., van Velzen, E., Palmateer, N., Kimber, J., Pharris, A., Hope, V., Taylor, A., Roy, K., Aspinall, E., Goldberg, D., Rhodes, T., Hedrich, D., Salminen, M., Hickman, M., & Hutchinson, S. J. (2014). Interventions to prevent HIV and hepatitis C in people who inject drugs: A review of reviews to assess evidence of effectiveness. *International Journal of Drug Policy*, *25*(1), 34–52. https://doi.org/10.1016/j.drugpo.2013.07.001

Macias-Konstantopoulos, W., Heins, A., Sachs, C. J., Whiteman, P. J., Wingkun, N.-J. G., & Riviello, R. J. (2021). Between emergency department visits: The role of harm reduction programs in mitigating the harms associated with injection drug use. *Annals of Emergency Medicine*, *77*(5), 479–492. https://doi.org/10.1016/j.annemergmed.2020.11.008

MacIsaac, M. B., Whitton, B., Hubble, A., Cogger, S., Penn, M., Weeks, A., Elmore, K., Pemberton, D., Anderson, J., Howard, R., McKeever, U., Papaluca, T., Hellard, M. E., Stoove, M., Wilson, D., Pedrana, A., Doyle, J., Clark, N., Holmes, J., & Thompson, A. J. (2023). Eliminating hepatitis C in Australia: a novel model of hepatitis C testing and treatment for people who inject drugs at a medically supervised injecting facility. *Medical Journal of Australia*, *218*(6), 256–261. https://doi.org/10.5694/mja2.51885

Madah-Amiri, D., Skulberg, A. K., Braarud, A.-C., Dale, O., Heyerdahl, F., Lobmaier, P., & Clausen, T. (2019). Ambulance-attended opioid overdoses: An examination into overdose locations and the role of a safe injection facility. *Substance Abuse*, *40*(3), 383–388. https://doi.org/10.1080/08897077.2018.1485130

Magwood, O., Salvalaggio, G., Beder, M., Kendall, C., Kpade, V., Daghmach, W., Habonimana, G., Marshall, Z., Snyder, E., O’Shea, T., Lennox, R., Hsu, H., Tugwell, P., & Pottie, K. (2020). The effectiveness of substance use interventions for homeless and vulnerably housed persons: A systematic review of systematic reviews on supervised consumption facilities, managed alcohol programs, and pharmacological agents for opioid use disorder. *PLoS ONE*, *15*(1), e0227298. <https://doi.org/10.1371/journal.pone.0227298>

Malkin, I. (2001). Establishing Supervised Injecting Facilities: A Responsible Way to Help Minimise Harm. *Melbourne University Law Review*, *25*(3), 680–756.

Mamdani, Z., McKenzie, S., Ackermann, E., Voyer, R., Cameron, F., Scott, T., Pauly, B., & Buxton, J. A. (2023). The cost of caring: Compassion fatigue among peer overdose response workers in British Columbia. *Substance Use and Misuse*, *58*(1), 85–93. https://doi.org/10.1080/10826084.2022.2148481

Markwick, N., Ti, L., Callon, C., Feng, C., Wood, E., & Kerr, T. (2014). Willingness to engage in peer-delivered HIV voluntary counselling and testing among people who inject drugs in a Canadian setting. *Journal of Epidemiology and Community Health*, *68*(7), 675–678. https://doi.org/10.1136/jech-2013-203707

Marshall, A. D., Grebely, J., Dore, G. J., & Treloar, C. (2017). ‘I didn’t want to let it go too far.’ The decisions and experiences of people who inject drugs who received a liver disease assessment as part of a liver health promotion campaign: The LiveRLife study. *International Journal of Drug Policy*, *47*, 153–160. https://doi.org/10.1016/j.drugpo.2017.06.001

Marshall, A. D., Micallef, M., Erratt, A., Telenta, J., Treloar, C., Everingham, H., Jones, S. C., Bath, N., How-Chow, D., Byrne, J., Harvey, P., Dunlop, A., Jauncey, M., Read, P., Collie, T., Dore, G. J., & Grebely, J. (2015). Liver disease knowledge and acceptability of non-invasive liver fibrosis assessment among people who inject drugs in the drug and alcohol setting: The LiveRLife Study. *International Journal of Drug Policy*, *26*(10), 984–991. https://doi.org/10.1016/j.drugpo.2015.07.002

Marshall, B. D. L., Wood, E., Zhang, R., Tyndall, M. W., Montaner, J. S. G., & Kerr, T. (2009). Condom use among injection drug users accessing a supervised injecting facility. *Sexually Transmitted Infections*, *85*(2), 121–126. https://doi.org/10.1136/sti.2008.032524

Marshall, B. D., Milloy, M.-J., Wood, E., Montaner, J. S., & Kerr, T. (2011). Reduction in overdose mortality after the opening of North America’s first medically supervised safer injecting facility: A retrospective population-based study. *The Lancet*, *377*(9775), 1429–1437. https://doi.org/10.1016/S0140-6736(10)62353-7

Marshall, B., & Wood, E. (2010). Toward a comprehensive approach to HIV prevention for people who use drugs. *Journal of Acquired Immune Deficiency Syndromes,* *55*, S23–S26. https://doi.org/10.1097/QAI.0b013e3181f9c203

Marshall, T., Abba-Aji, A., Tanguay, R., & Greenshaw, A. J. (2021). The impact of supervised consumption services on fentanyl-related deaths: Lessons learned from Alberta’s provincial data. *Canadian Journal of Psychiatry*, *66*(12), 1096–1098. https://doi.org/10.1177/0706743721999571

Mathis, S., Hagemeier, N., Hagaman, A., Dreyzehner, J., & Pack, R. (2018). A dissemination and implementation science approach to the epidemic of opioid use disorder in the United States. *Current HIV/AIDS Reports*, *15*(5), 359–370. https://doi.org/10.1007/s11904-018-0409-9

May, T. (2017). Medically Supervised Injecting Centres (MSICs): A review of systematic reviews. Report submitted to the Welsh Government’s Advisory Panel on Substance Misuse (APoSM). Available from: <https://www.gov.wales/sites/default/files/publications/2019-01/180320atisn12038doc2_0.pdf>

Mayer, S., Boyd, J., Collins, A., Kennedy, M. C., Fairbairn, N., & McNeil, R. (2018). Characterizing fentanyl-related overdoses and implications for overdose response: Findings from a rapid ethnographic study in Vancouver, Canada. *Drug and Alcohol Dependence*, *193*, 69–74. https://doi.org/10.1016/j.drugalcdep.2018.09.006

McCann, M., & Vadivelu, S. (2019). Saving lives. Changing lives. Summary report on the findings from an evaluation of London’s temporary overdose prevention site (TOPS), Ontario. Available from: <https://static1.squarespace.com/static/599320d3b8a79baf4289fc66/t/5d7a646ddbcfcb27b1171f21/1568302202798/TOPSsummary_2019-04-08.pdf>

McGinty, E. E., Barry, C. L., Stone, E. M., Niederdeppe, J., Kennedy-Hendricks, A., Linden, S., & Sherman, S. G. (2018). Public support for safe consumption sites and syringe services programs to combat the opioid epidemic. *Preventive Medicine*, *111*, 73–77. https://doi.org/10.1016/j.ypmed.2018.02.026

McKnight, I., Maas, B., Wood, E., Tyndall, M. W., Small, W., Lai, C., Montaner, J. S. G., & Kerr, T. (2007). Factors associated with public injecting among users of Vancouver’s supervised injection facility. *American Journal of Drug and Alcohol Abuse*, *33*(2), 319–325. https://doi.org/10.1080/00952990601175102

McNeil, R., Dilley, L. B., Guirguis-Younger, M., Hwang, S. W., & Small, W. (2014). Impact of supervised drug consumption services on access to and engagement with care at a palliative and supportive care facility for people living with HIV/AIDS: A qualitative study. *Journal of the International AIDS Society*, *17*. https://doi.org/10.7448/IAS.17.1.18855

McNeil, R., Kerr, T., Lampkin, H., & Small, W. (2015). “We need somewhere to smoke crack”: An ethnographic study of an unsanctioned safer smoking room in Vancouver, Canada. *International Journal of Drug Policy*, *26*(7), 645–652. https://doi.org/10.1016/j.drugpo.2015.01.015

McNeil, R., Shannon, K., Shaver, L., Kerr, T., & Small, W. (2014). Negotiating place and gendered violence in Canada’s largest open drug scene. *International Journal of Drug Policy*, *25*(3), 608–615. https://doi.org/10.1016/j.drugpo.2013.11.006

McNeil, R., & Small, W. (2014). “Safer environment interventions”: A qualitative synthesis of the experiences and perceptions of people who inject drugs. *Social Science and Medicine*, *106*, 151–158. https://doi.org/10.1016/j.socscimed.2014.01.051

McNeil, R., Small, W., Lampkin, H., Shannon, K., & Kerr, T. (2014). “People knew they could come here to get help”: An ethnographic study of assisted injection practices at a peer-run ‘unsanctioned’ supervised drug consumption room in a Canadian setting. *AIDS and Behavior*, *18*(3), 473–485. <https://doi.org/10.1007/s10461-013-0540-y>

Medically Supervised Injecting Room Review Panel. (2020). Review of the Medically Supervised Injecting Room. Available from: <https://www.health.vic.gov.au/aod-treatment-services/review-of-the-medically-supervised-injecting-room-trial>

Mema, S. C., Frosst, G., Bridgeman, J., Drake, H., Dolman, C., Lappalainen, L., & Corneil, T. (2019). Mobile supervised consumption services in Rural British Columbia: lessons learned. *Harm Reduction Journal*, *16*(4), 4. https://doi.org/10.1186/s12954-018-0273-3

Mercer, F., Miler, J. A., Pauly, B., Carver, H., Hnízdilová, K., Foster, R., & Parkes, T. (2021). Peer support and overdose prevention responses: a systematic ‘state-of-the-art’review. *International Journal of Environmental Research and Public Health*, *18*(22). https://doi.org/10.3390/ijerph182212073

Miller, N. M., Waterhouse-Bradley, B., Campbell, C., & Shorter, G. W. (2022). How do naloxone-based interventions work to reduce overdose deaths: a realist review. *Harm Reduction Journal*, *19*(1), 1–13.

Milloy, M.-J. S., Kerr, T., Mathias, R., Zhang, R., Montaner, J. S., Tyndall, M., & Wood, E. (2008). Non-fatal overdose among a cohort of active injection drug users recruited from a supervised injection facility. *American Journal of Drug and Alcohol Abuse*, *34*(4), 499–509. https://doi.org/10.1080/00952990802122457

Milloy, M.-J. S., Kerr, T., Tyndall, M., Montaner, J., & Wood, E. (2008). Estimated drug overdose deaths averted by North America’s first medically-supervised safer injection facility. *PLoS ONE*, *3*(10). https://doi.org/10.1371/journal.pone.0003351

Milloy, M.-J. S., Kerr, T., Zhang, R., Tyndall, M., Montaner, J., & Wood, E. (2010). Inability to access addiction treatment and risk of HIV infection among injection drug users recruited from a supervised injection facility. *Journal of Public Health*, *32*(3), 342–349. https://doi.org/10.1093/pubmed/fdp089

Milloy, M.-J., Wood, E., Tyndall, M., Lai, C., Montaner, J., & Kerr, T. (2009). Recent incarceration and use of a supervised injection facility in Vancouver, Canada. *Addiction Research and Theory*, *17*(5), 538–545. https://doi.org/10.1080/16066350802023065

Mitra, S., Rachlis, B., Krysowaty, B., Marshall, Z., Olsen, C., Rourke, S., & Kerr, T. (2019). Potential use of supervised injection services among people who inject drugs in a remote and mid-size Canadian setting. *BMC Public Health*, *19*(1). https://doi.org/10.1186/s12889-019-6606-7

Mitra, S., Rachlis, B., Scheim, A., Bardwell, G., Rourke, S. B., & Kerr, T. (2017). Acceptability and design preferences of supervised injection services among people who inject drugs in a mid-sized Canadian City. *Harm Reduction Journal*, *14*(1). https://doi.org/10.1186/s12954-017-0174-x

Montero-Moraga, J. M., Garrido-Albaina, A., Barbaglia, M. G., Gotsens, M., Aranega, D., Espelt, A., & Parés-Badell, O. (2020). Impact of 24-hour schedule of a drug consumption room on service use and number of non-fatal overdoses. A quasi-experimental study in Barcelona. *International Journal of Drug Policy*, *81*. https://doi.org/10.1016/j.drugpo.2020.102772

Moshkforoush, M., DeBeck, K., Brar, R., Fairbairn, N., Cui, Z., Milloy, M.-J., Buxton, J., Oldenburger, T., McLellan, W., Kendall, P., Sedgemore, K., Wilson, D., Kerr, T., & Hayashi, K. (2022). Low awareness of risk mitigation prescribing in response to dual crises of COVID-19 and overdose deaths among people who use unregulated drugs in Vancouver, Canada. *Harm Reduction Journal*, *19*(1). https://doi.org/10.1186/s12954-022-00632-6

Mrazovac, A., O’Boyle, J., Watts, C., Sharma, T., Ciccarelli, M., Leshuk, T., Lachhman, R., Michael, S., & Manwell, L. (2020). Public knowledge of and support for supervised injection sites in a metropolitan Canadian region. *International Journal of Mental Health and Addiction*, *18*(1), 236–256. https://doi.org/10.1007/s11469-019-00130-0

MSIC Evaluation Committee. (2003). Final report of the evaluation of the Sydney medically supervised injecting centre. Available from: <https://www.drugsandalcohol.ie/5706/1/MSIC_final_evaluation_report.pdf>

Munoz Sastre, M. T., Kpanake, L., & Mullet, E. (2020). French People’s positions on supervised injection facilities for drug users. *Substance Abuse: Treatment, Prevention, and Policy*, *15*(1). https://doi.org/10.1186/s13011-020-00321-2

Myer, A. J., & Belisle, L. (2018). Highs and lows: An interrupted time-series evaluation of the impact of North America’s only supervised injection facility on crime. *Journal of Drug Issues*, *48*(1), 36–49. https://doi.org/10.1177/0022042617727513

Nassau, T., Kolla, G., Mason, K., Hopkins, S., Tookey, P., McLean, E., Werb, D., & Scheim, A. (2022). Service utilization patterns and characteristics among clients of integrated supervised consumption sites in Toronto, Canada. *Harm Reduction Journal*, *19*(33), 33. https://doi.org/10.1186/s12954-022-00610-y

Navarro, C., & Leonard, L. (2004). Prevalence and factors related to public injecting in Ottawa, Canada: Implications for the development of a trial safer injecting facility. *International Journal of Drug Policy*, *15*(4), 275–284. https://doi.org/10.1016/j.drugpo.2004.03.003

Nielsen, S., Barratt, M., Hiley, S., Bartlett, M., Latimer, J., Jauncey, M., Roux, C., Morelato, M., Clark, N., Kowalski, M., Gilbert, M., Francia, L., Shipton, A., Gerostamoulos, D., Glowacki, L., & Lam, T. (2023). Monitoring for fentanyl within Australian supervised injecting facilities: Findings from feasibility testing of novel methods and collaborative workshops. *International Journal of Drug Policy*, *115*. https://doi.org/10.1016/j.drugpo.2023.104015

Nolan, S., Kelian, S., Kerr, T., Young, S., Malmgren, I., Ghafari, C., Harrison, S., Wood, E., Lysyshyn, M., & Holliday, E. (2022). Harm reduction in the hospital: An overdose prevention site (OPS) at a Canadian hospital. *Drug and Alcohol Dependence*, *239*. https://doi.org/10.1016/j.drugalcdep.2022.109608

Notta, D., Black, B., Chu, T., Joe, R., & Lysyshyn, M. (2019). Changing risk and presentation of overdose associated with consumption of street drugs at a supervised injection site in Vancouver, Canada. *Drug and Alcohol Dependence*, *196*, 46–50. https://doi.org/10.1016/j.drugalcdep.2018.12.016

O’Rourke, A., White, R. H., Park, J. N., Rodriguez, K., Kilkenny, M. E., Sherman, S. G., & Allen, S. T. (2019). Acceptability of safe drug consumption spaces among people who inject drugs in rural West Virginia. *Harm Reduction Journal*, *16*(1). https://doi.org/10.1186/s12954-019-0320-8

Olding, M., Boyd, J., Kerr, T., Fowler, A., & McNeil, R. (2023). (Re)situating expertise in community-based overdose response: Insights from an ethnographic study of overdose prevention sites (OPS) in Vancouver, Canada. *International Journal of Drug Policy*, *111*. https://doi.org/10.1016/j.drugpo.2022.103929

Olding, M., Boyd, J., Kerr, T., & McNeil, R. (2021). “And we just have to keep going”: Task shifting and the production of burnout among overdose response workers with lived experience. *Social Science and Medicine*, *270*. https://doi.org/10.1016/j.socscimed.2020.113631

Olding, M., Ivsins, A., Mayer, S., Betsos, A., Boyd, J., Sutherland, C., Culbertson, C., Kerr, T., & McNeil, R. (2020). A low-barrier and comprehensive community-based harm-reduction site in Vancouver, Canada. *American Journal of Public Health*, *110*(6), 833–835. https://doi.org/10.2105/AJPH.2020.305612

Otter, D. (2016). Safe Consumption Facilities: Evidence and Models. Available from: <https://kingcounty.gov/~/media/depts/community-human-services/behavioral-health-recovery/documents/herointf/safe_consumption_facilities_evidence_models.ashx?la=en>

Oudshoorn, A., Sangster Bouck, M., McCann, M., Zendo, S., Berman, H., Banninga, J., Le Ber, M. J., & Zendo, Z. (2021). A critical narrative inquiry to understand the impacts of an overdose prevention site on the lives of site users. *Harm Reduction Journal*, *18*(1). https://doi.org/10.1186/s12954-020-00458-0

Panagiotoglou, D. (2022). Evaluating the population-level effects of overdose prevention sites and supervised consumption sites in British Columbia, Canada: Controlled interrupted time series. *PLoS ONE*, *17*(3). https://doi.org/10.1371/journal.pone.0265665

Panagiotoglou, D., & Lim, J. (2022). Using synthetic controls to estimate the population-level effects of Ontario’s recently implemented overdose prevention sites and consumption and treatment services. *International Journal of Drug Policy*, *110*. https://doi.org/10.1016/j.drugpo.2022.103881

Pardo, B., Kilmer, B., & Caulkins, J. P. (2018). Assessing the evidence on supervised drug consumption sites. Available from: <https://www.rand.org/pubs/working_papers/WR1261.html>

Park, J. N., Sherman, S. G., Rouhani, S., Morales, K. B., McKenzie, M., Allen, S. T., Marshall, B. D. L., & Green, T. C. (2019). Willingness to use safe consumption spaces among opioid users at high risk of fentanyl overdose in Baltimore, Providence, and Boston. *Journal of Urban Health*, *96*(3), 353–366. https://doi.org/10.1007/s11524-019-00365-1

Parkes, T., Price, T., Foster, R., Trayner, K. M. A., Sumnall, H. R., Livingston, W., Perkins, A., Cairns, B., Dumbrell, J., & Nicholls, J. (2022). ‘Why would we not want to keep everybody safe?’ The views of family members of people who use drugs on the implementation of drug consumption rooms in Scotland. *Harm Reduction Journal*, *19*(1). https://doi.org/10.1186/s12954-022-00679-5

Patterson, T., Bharmal, A., Padhi, S., Buchner, C., Gibson, E., & Lee, V. (2018). Opening Canada’s first Health Canada-approved supervised consumption sites. *Canadian Journal of Public Health*, *109*(4), 581–584. https://doi.org/10.17269/s41997-018-0107-9

Pauly, B., Wallace, B., Pagan, F., Phillips, J., Wilson, M., Hobbs, H., & Connolly, J. (2020). Impact of overdose prevention sites during a public health emergency in Victoria, Canada. *PLoS ONE*, *15*(5). https://doi.org/10.1371/journal.pone.0229208

Paumier, R. (2022). Between zero risk and harm reduction: An ethnography of Montreal supervised injection services as a public policy instrument. *International Journal of Drug Policy*, *104*. https://doi.org/10.1016/j.drugpo.2022.103694

Peacey, J. (2014). Drug consumption rooms in Europe: client experience survey in Amsterdam and Rotterdam. Available from: <https://www.academia.edu/9215441/Drug_Consumption_Rooms_in_Europe_Client_experience_survey_in_Amsterdam_and_Rotterdam>

Pennington, M. L., Dupree, J., Coe, E., Ostiguy, W., Kimbrel, N. A., Meyer, E. C., & Gulliver, S. B. (2021). Working near a supervised injection facility: A qualitative study of perspectives of firefighter-emergency medical responders. *American Journal of Industrial Medicine*, *64*(4), 296–300. https://doi.org/10.1002/ajim.23224

Petrar, S., Kerr, T., Tyndall, M. W., Zhang, R., Montaner, J. S. G., & Wood, E. (2007). Injection drug users’ perceptions regarding use of a medically supervised safer injecting facility. *Addictive Behaviors*, *32*(5), 1088–1093. https://doi.org/10.1016/j.addbeh.2006.07.013

Philbin, M. M., Lozada, R., Zúñiga, M. L., Mantsios, A., Case, P., Magis-Rodriguez, C., Latkin, C. A., & Strathdee, S. A. (2008). A qualitative assessment of stakeholder perceptions and socio-cultural influences on the acceptability of harm reduction programs in Tijuana, Mexico. *Harm Reduction Journal*, *5*. https://doi.org/10.1186/1477-7517-5-36

Philbin, M. M., Mantsios, A., Lozada, R., Case, P., Pollini, R. A., Alvelais, J., Latkin, C. A., Magis-Rodriguez, C., & Strathdee, S. A. (2009). Exploring stakeholder perceptions of acceptability and feasibility of needle exchange programmes, syringe vending machines and safer injection facilities in Tijuana, Mexico. *International Journal of Drug Policy*, *20*(4), 329–335. https://doi.org/10.1016/j.drugpo.2008.09.002

Pijl, E., Oosterbroek, T., Motz, T., Mason, E., & Hamilton, K. (2021). Peer-assisted injection as a harm reduction measure in a supervised consumption service: a qualitative study of client experiences. *Harm Reduction Journal*, *18*(1). https://doi.org/10.1186/s12954-020-00455-3

Pinkerton, S. D. (2010). Is Vancouver Canada’s supervised injection facility cost-saving? *Addiction*, *105*(8), 1429–1436. https://doi.org/10.1111/j.1360-0443.2010.02977.x

Potier, C., Laprévote, V., Dubois-Arber, F., Cottencin, O., & Rolland, B. (2014). Supervised injection services: What has been demonstrated? A systematic literature review. *Drug and Alcohol Dependence*, *145*, 48–68. https://doi.org/10.1016/j.drugalcdep.2014.10.012

Power, J., Salmon, A. M., Latimer, J., Jauncey, M., & Day, C. A. (2019). Overdose risk and client characteristics associated with the injection of buprenorphine at a medically supervised injecting center in Sydney, Australia. *Substance Use and Misuse*, *54*(10), 1646–1653. https://doi.org/10.1080/10826084.2019.1600147

Prangnell, A., Fairgrieve, C., Nosova, E., DeBeck, K., Milloy, M.-J., & Hayashi, K. (2019). High prevalence of self-reported exposure to adulterated drugs among people who experienced an opioid overdose in Canada: A cohort study. *Substance Use and Misuse*, *54*(6), 980–985. https://doi.org/10.1080/10826084.2018.1555257

Rammohan, I., Bouck, Z., Fusigboye, S., Bowles, J., McDonald, K., Maghsoudi, N., Scheim, A., & Werb, D. (2022). Drug checking use and interest among people who inject drugs in Toronto, Canada. *International Journal of Drug Policy*, *107*. https://doi.org/10.1016/j.drugpo.2022.103781

Rance, J., & Fraser, S. (2011). Accidental intimacy: transformative emotion and the Sydney Medically Supervised Injecting Centre. *Contemporary Drug Problems*, *38*(1), 121–145. https://doi.org/10.1177/009145091103800106

Reddon, H., Marshall, B. D. L., & Milloy, M.-J. (2019). Elimination of HIV transmission through novel and established prevention strategies among people who inject drugs. *The Lancet HIV*, *6*(2), e128–e136. https://doi.org/10.1016/S2352-3018(18)30292-3

Reddon, H., Wood, E., Tyndall, M., Lai, C., Hogg, R., Montaner, J., & Kerr, T. (2011). Use of North America’s first medically supervised safer injecting facility among HIV-positive injection drug users. *AIDS Education and Prevention*, *23*(5), 412–422. https://doi.org/10.1521/aeap.2011.23.5.412

Richardson, L., Wood, E., Zhang, R., Montaner, J., Tyndall, M., & Kerr, T. (2008). Employment among users of a medically supervised safer injection facility. *American Journal of Drug and Alcohol Abuse*, *34*(5), 519–525. https://doi.org/10.1080/00952990802146308

Rickard, G., & Hart, B. (2022). Survival, safety and belonging: An ethnographic study of experiences and perceptions of people who inject drugs accessing a supervised injecting Centre. *Australian Journal of Social Issues*, *57*(4), 829–846. https://doi.org/10.1002/ajs4.230

Ritter, A., & Cameron, J. (2006). A review of the efficacy and effectiveness of harm reduction strategies for alcohol, tobacco and illicit drugs. *Drug and Alcohol Review*, *25*(6), 611–624. https://doi.org/10.1080/09595230600944529

Roncero, C., Martínez-Luna, N., Daigre, C., Grau-López, L., Gonzalvo, B., Pérez-Pazos, J., & Casas, M. (2013). Psychotic symptoms of cocaine self-injectors in a harm reduction program. *Substance Abuse*, *34*(2), 118–121. https://doi.org/10.1080/08897077.2012.691446

Roth, A. M., Kral, A. H., Mitchell, A., Mukherjee, R., Davidson, P., & Lankenau, S. E. (2019). Overdose prevention site acceptability among residents and businesses surrounding a proposed site in Philadelphia, USA. *Journal of Urban Health*. https://doi.org/10.1007/s11524-019-00364-2

Rouhani, S., White, R. H., Park, J. N., & Sherman, S. G. (2020). High willingness to use overdose prevention sites among female sex workers in Baltimore, Maryland. *Drug and Alcohol Dependence*, *212*. https://doi.org/10.1016/j.drugalcdep.2020.108042

Roux, P., Jauffret-Roustide, M., Donadille, C., Madrid, L., Denis, C., Celerier, I., Chauvin, C., Hamelin, N., Maradan, G., Carrieri, M., Protopopescu, C., Lalanne, L., Auriacombe, M., & Grp, C. S. (2023). Impact of drug consumption rooms on non-fatal overdoses, abscesses and emergency department visits in people who inject drugs in France: results from the COSINUS cohort. *International Journal of Epidemiology.* https://doi.org/10.1093/ije/dyac120

Rowe, A., Chang, A., Lostchuck, E., Lin, K., Scheuermeyer, F., McCann, V., Moe, J., Cho, R., Clerc, P., McSweeney, C., Jiang, A., & Purssell, R. (2022). Out-of-hospital management of unresponsive, apnoeic, witnessed opioid overdoses: a case series from a supervised consumption site. *Canadian Journal of Emergency Medicine*, *24*(6), 650–658. https://doi.org/10.1007/s43678-022-00326-9

Roxburgh, A., Darke, S., Salmon, A. M., Dobbins, T., & Jauncey, M. (2017). Frequency and severity of non-fatal opioid overdoses among clients attending the Sydney Medically Supervised Injecting Centre. *Drug and Alcohol Dependence*, *176*, 126–132. https://doi.org/10.1016/j.drugalcdep.2017.02.027

Roxburgh, A., Jauncey, M., Day, C., Bartlett, M., Cogger, S., Dietze, P., Nielsen, S., Latimer, J., & Clark, N. (2021). Adapting harm reduction services during COVID-19: lessons from the supervised injecting facilities in Australia. *Harm Reduction Journal*, *18*(1). https://doi.org/10.1186/s12954-021-00471-x

Rudzinski, K., Xavier, J., Guta, A., Chan Carusone, S., King, K., Phillips, J. C., Switzer, S., O’Leary, B., Baltzer Turje, R., Harrison, S., de Prinse, K., Simons, J., & Strike, C. (2021). Feasibility, acceptability, concerns, and challenges of implementing supervised injection services at a specialty HIV hospital in Toronto, Canada: perspectives of people living with HIV. *BMC Public Health*, *21*(1). <https://doi.org/10.1186/s12889-021-11507-z>

Salmon, A., Belackova, V., Schwanz, R. S., Jauncey, M., Hiley, S., & Demirkol, A. (2017). Homelessness among clients of Sydney’s supervised injecting facility. *Drugs and Alcohol Today*, *17*(4), 258–268. https://doi.org/10.1108/DAT-06-2017-0026

Salmon, A. M., Dwyer, R., Jauncey, M., van Beek, I., Topp, L., & Maher, L. (2009). Injecting-related injury and disease among clients of a supervised injecting facility. *Drug and Alcohol Dependence*, *101*(1), 132–136. https://doi.org/10.1016/j.drugalcdep.2008.12.002

Salmon, A. M., Thein, H.-H., Kimber, J., Kaldor, J. M., & Maher, L. (2007). Five years on: What are the community perceptions of drug-related public amenity following the establishment of the Sydney Medically Supervised Injecting Centre? *International Journal of Drug Policy*, *18*(1), 46–53. https://doi.org/10.1016/j.drugpo.2006.11.010

Salmon, A. M., Van Beek, I., Amin, J., Grulich, A., & Maher, L. (2009). High HIV testing and low HIV prevalence among injecting drug users attending the Sydney Medically Supervised Injecting Centre. *Australian and New Zealand Journal of Public Health*, *33*(3), 280–283. https://doi.org/10.1111/j.1753-6405.2009.00389.x

Salmon, A. M., Van Beek, I., Amin, J., Kaldor, J., & Maher, L. (2010). Study on drug consumption rooms on current practice and future capacity to address communicable diseases like HCV. *Addiction*, *105*(4), 676–683. https://doi.org/10.1111/j.1360-0443.2009.02837.x

Scheim, A. I., Bouck, Z., Tookey, P., Hopkins, S., Sniderman, R., McLean, E., Garber, G., Baral, S., Rourke, S. B., & Werb, D. (2021). Supervised consumption service use and recent non-fatal overdose among people who inject drugs in Toronto, Canada. *International Journal of Drug Policy*, *87*. https://doi.org/10.1016/j.drugpo.2020.102993

Scheim, A. I., Sniderman, R., Wang, R., Bouck, Z., McLean, E., Mason, K., Bardwell, G., Mitra, S., Greenwald, Z. R., Thavorn, K., Garber, G., Baral, S. D., Rourke, S. B., & Werb, D. (2021). The Ontario integrated supervised injection services cohort study of people who inject drugs in Toronto, Canada (OiSIS-Toronto): cohort profile. *Journal of Urban Health*, *98*(4), 538–550. https://doi.org/10.1007/s11524-021-00547-w

Scherbaum, N., Specka, M., Bombeck, J., & Marrziniak, B. (2009). Drug consumption facility as part of a primary health care centre for problem drug users-Which clients are attracted? *International Journal of Drug Policy*, *20*(5), 447–449. https://doi.org/10.1016/j.drugpo.2009.01.001

Scherbaum, N., Specka, M., Schifano, F., Bombeck, J., & Marrziniak, B. (2010). Longitudinal observation of a sample of German drug consumption facility clients. *Substance Use and Misuse*, *45*(1), 176–189. https://doi.org/10.3109/10826080902873044

Scherbaum, N., Timm, J., Richter, F., Bonnet, U., Bombeck, J., Lajos, S., & Specka, M. (2018). Outcome of a hepatitis B vaccination program for clients of a drug consumption facility. *Journal of Clinical Virology*, *106*, 28–32. https://doi.org/10.1016/j.jcv.2018.04.014

Schulte, B., Schmidt, C. S., Strada, L., Götzke, C., Hiller, P., Fischer, B., & Reimer, J. (2016). Non-prescribed use of opioid substitution medication: Patterns and trends in sub-populations of opioid users in Germany. *International Journal of Drug Policy*, *29*, 57–65. https://doi.org/10.1016/j.drugpo.2015.12.024

Semaan, S., Fleming, P., Worrell, C., Stolp, H., Baack, B., & Miller, M. (2011). Potential role of safer injection facilities in reducing HIV and Hepatitis C infections and overdose mortality in the United States. *Drug and Alcohol Dependence*, *118*(2), 100–110. https://doi.org/10.1016/j.drugalcdep.2011.03.006

Shaw, A., Lazarus, L., Pantalone, T., LeBlanc, S., Lin, D., Stanley, D., Chepesiuk, C., Patel, S., Tyndall, M., Kelly, F., Chris, D., Dan, M., Rick, S., Tyler, P., Hana, D., Alana, M., Tarah, H., Caleb, C., Fred, C., … & Sharp, D. (2015). Risk environments facing potential users of a supervised injection site in Ottawa, Canada. *Harm Reduction Journal*, *12*(1), 1. https://doi.org/10.1186/s12954-015-0083-9

Sherman, S. G., Rouhani, S., White, R. H., Weicker, N., Morris, M., Schneider, K., Park, J. N., & Barry, C. (2022). Acceptability of overdose prevention sites in the business community in Baltimore, Maryland. *Journal of Urban Health*, *99*(4), 723–732. https://doi.org/10.1007/s11524-022-00647-1

Shorter, G. W., Harris, M., McAuley, A., Trayner, K. M., & Stevens, A. (2022). The United Kingdom’s first unsanctioned overdose prevention site; A proof-of-concept evaluation. *International Journal of Drug Policy*, *104*. https://doi.org/10.1016/j.drugpo.2022.103670

Skelton, E., Tzelepis, F., Shakeshaft, A., Guillaumier, A., Wood, W., Jauncey, M., Salmon, A. M., McCrabb, S., & Bonevski, B. (2019). Integrating smoking cessation care into a medically supervised injecting facility using an organizational change intervention: A qualitative study of staff and client views. *International Journal of Environmental Research and Public Health*, *16*(11). https://doi.org/10.3390/ijerph16112050

Skelton, E., Tzelepis, F., Shakeshaft, A., Guillaumier, A., Wood, W., Jauncey, M., Salmon, A. M., McCrabb, S., Palazzi, K., & Bonevski, B. (2018). Integrating smoking cessation care into routine service delivery in a medically supervised injecting facility: An acceptability study. *Addictive Behaviors*, *84*, 193–200. https://doi.org/10.1016/j.addbeh.2018.04.001

Small, W., Ainsworth, L., Wood, E., & Kerr, T. (2011). IDU perspectives on the design and operation of north America’s first medically supervised injection facility. *Substance Use and Misuse*, *46*(5), 561–568. https://doi.org/10.3109/10826084.2010.517714

Small, W., Krusi, A., Wood, E., Montaner, J., & Kerr, T. (2012). Street-level policing in the Downtown Eastside of Vancouver, Canada, during the 2010 winter Olympics. *International Journal of Drug Policy*, *23*(2), 128–133. https://doi.org/10.1016/j.drugpo.2011.06.007

Small, W., Moore, D., Shoveller, J., Wood, E., & Kerr, T. (2012). Perceptions of risk and safety within injection settings: Injection drug users’ reasons for attending a supervised injecting facility in Vancouver, Canada. *Health, Risk and Society*, *14*(4), 307–324. https://doi.org/10.1080/13698575.2012.680950

Small, W., Shoveller, J., Moore, D., Tyndall, M., Wood, E., & Kerr, T. (2011). Injection drug users’ access to a supervised injection facility in Vancouver, Canada: The influence of operating policies and local drug culture. *Qualitative Health Research*, *21*(6), 743–756. https://doi.org/10.1177/1049732311400919

Small, W., Van Borek, N., Fairbairn, N., Wood, E., & Kerr, T. (2009). Access to health and social services for IDU: The impact of a medically supervised injection facility. *Drug and Alcohol Review*, *28*(4), 341–346. https://doi.org/10.1111/j.1465-3362.2009.00025.x

Small, W., Wood, E., Lloyd-Smith, E., Tyndall, M., & Kerr, T. (2008). Accessing care for injection-related infections through a medically supervised injecting facility: A qualitative study. *Drug and Alcohol Dependence*, *98*(1), 159–162. https://doi.org/10.1016/j.drugalcdep.2008.05.014

Smith, P., Favril, L., Delhauteur, D., Vander Laenen, F., & Nicaise, P. (2019). How to overcome political and legal barriers to the implementation of a drug consumption room: An application of the policy agenda framework to the Belgian situation. *Addiction Science and Clinical Practice*, *14*(1). https://doi.org/10.1186/s13722-019-0169-x

Socia, K. M., Stone, R., Palacios, W. R., & Cluverius, J. (2021). Focus on prevention: The public is more supportive of “overdose prevention sites” than they are of “safe injection facilities.” *Criminology and Public Policy*, *20*(4), 729–754. https://doi.org/10.1111/1745-9133.12566

Solai, S., Dubois-Arber, F., Benninghoff, F., & Benaroyo, L. (2006). Ethical reflections emerging during the activity of a low threshold facility with supervised drug consumption room in Geneva, Switzerland. *International Journal of Drug Policy*, *17*(1), 17–22. https://doi.org/10.1016/j.drugpo.2005.12.008

Southwell, M., Scher, B., Harris, M., & Shorter, G. W. (2022). The Case for Overdose Prevention Centres: Voices from Sandwell. Available: <https://pure.qub.ac.uk/en/publications/the-case-for-overdose-prevention-centres-voices-from-sandwell>

Speed, K. A., Gehring, N. D., Launier, K., O’Brien, D., Campbell, S., & Hyshka, E. (2020). To what extent do supervised drug consumption services incorporate non-injection routes of administration: A systematic scoping review documenting existing facilities. *Harm Reduction Journal*, *17*(1). https://doi.org/10.1186/s12954-020-00414-y

Stam, N. C., Cogger, S., Schumann, J. L., Weeks, A., Roxburgh, A., Dietze, P. M., & Clark, N. (2022). The onset and severity of acute opioid toxicity in heroin overdose cases: a retrospective cohort study at a supervised injecting facility in Melbourne, Australia. *Clinical Toxicology*, *60*(11), 1227–1234. https://doi.org/10.1080/15563650.2022.2126371

Steele, M., Silins, E., Flaherty, I., Hiley, S., van Breda, N., & Jauncey, M. (2018). Uptake of wheel-filtration among clients of a supervised injecting facility: Can structured education work? *Drug and Alcohol Review*, *37*(1), 116–120. https://doi.org/10.1111/dar.12481

Stoever, H. (2002). Consumption rooms - A middle ground between health and public order concerns. *Journal of Drug Issues*, *32*(2), 597–606. https://doi.org/10.1177/002204260203200217

Stoltz, J.A. M., Wood, E., Miller, C., Small, W., Li, K., Tyndall, M., Montaner, J., & Kerr, T. (2007). Characteristics of young illicit drug injectors who use North America’s first medically supervised safer injecting facility. *Addiction Research and Theory*, *15*(1), 63–69. https://doi.org/10.1080/16066350601081090

Stoltz, J. A. M., Wood, E., Small, W., Li, K., Tyndall, M., Montaner, J., & Kerr, T. (2007). Changes in injecting practices associated with the use of a medically supervised safer injection facility. *Journal of Public Health*, *29*(1), 35–39. https://doi.org/10.1093/pubmed/fdl090

Stöver, H., & Förster, S. (2020). Drug Consumption Rooms (DCRs) in Frankfurt am Main/Germany. Annual Report of the Monitoring of 4 DCRs in 2019. Available from: <https://www.frankfurt-university.de/fileadmin/standard/Hochschule/Fachbereich_4/Forschung/ISFF/Forschungsprojekte/Jahresbericht_2020_engl_KW1.7.2020_final_1_.pdf>

Stöver, H. J., & Schäffer, D. (2014). Smoke it! Promoting a change of opiate consumption pattern - From injecting to inhaling. *Harm Reduction Journal*, *11*(1). https://doi.org/10.1186/1477-7517-11-18

Strike, C., Jairam, J. A., Kolla, G., Millson, P., Shepherd, S., Fischer, B., Watson, T. M., & Bayoumi, A. M. (2014). Increasing public support for supervised injection facilities in Ontario, Canada. *Addiction*, *109*(6), 946–953. https://doi.org/10.1111/add.12506

Strike, C., Rotondi, N. K., Watson, T. M., Kolla, G., & Bayoumi, A. M. (2016). Public opinions about supervised smoking facilities for crack cocaine and other stimulants. *Substance Abuse: Treatment, Prevention, and Policy*, *11*(1). https://doi.org/10.1186/s13011-016-0052-7

Strike, C., Watson, T. M., Altenberg, J., Barnaby, L., Bayoumi, A. M., Challacombe, L., Demel, G., Hopkins, S., & Wright, A. (2020). Challenges, skepticism, and recommendations from police about working in collaboration with supervised consumption services. *Substance Use and Misuse*, 1919–1924. https://doi.org/10.1080/10826084.2020.1781177

Strike, C., Watson, T. M., Kolla, G., Penn, R., & Bayoumi, A. M. (2015). Ambivalence about supervised injection facilities among community stakeholders. *Harm Reduction Journal*, *12*(1). https://doi.org/10.1186/s12954-015-0060-3

Suen, L. W., Davidson, P. J., Browne, E. N., Lambdin, B. H., Wenger, L. D., & Kral, A. H. (2022). Effect of an unsanctioned safe consumption site in the United States on syringe sharing, rushed injections, and isolated injection drug use: A longitudinal cohort analysis. *Journal of Acquired Immune Deficiency Syndromes*, *89*(2), 172–177. https://doi.org/10.1097/QAI.0000000000002849

Sumnall, H. R., Atkinson, A. M., Trayner, K. M. A., Gage, S. H., & McAuley, A. (2020). Effects of messaging on public support for drug consumption rooms in Scotland, UK. *International Journal of Drug Policy*, *83*. https://doi.org/10.1016/j.drugpo.2020.102855

Sylvester, S. M., Haeder, S. F., & Callaghan, T. (2022). Just say no? Public attitudes about supportive and punitive policies to combat the opioid epidemic. *Journal of Public Policy*, *42*(2), 270–297. https://doi.org/10.1017/S0143814X21000155

Tammi, T., Rigoni, R., Maticic, M., Schäffer, D., Perez Gayo, R., & Schatz, E. (2020). Drug Consumption Rooms: Civil Society Monitoring of Harm Reduction in Europe, 2019. Data Report. 78–82. Available from: <https://www.correlation-net.org/wp-content/uploads/2021/03/monitoring_report2020.pdf>

Taylor, H., Curado, A., Tavares, J., Oliveira, M., Gautier, D., & Maria, J. S. (2019). Prospective client survey and participatory process ahead of opening a mobile drug consumption room in Lisbon. *Harm Reduction Journal*, *16*(1). https://doi.org/10.1186/s12954-019-0319-1

Taylor, H., Leite, Â., Gautier, D., Nunes, P., Pires, J., & Curado, A. (2022). Community perceptions surrounding Lisbon’s first mobile drug consumption room. *Dialogues in Health*, *1*. https://doi.org/10.1016/j.dialog.2022.100031

Taylor, J., Ober, A. J., Kilmer, B., Caulkins, J. P., & Iguchi, M. Y. (2021). Community perspectives on supervised consumption sites: Insights from four U.S. counties deeply affected by opioids. *Journal of Substance Abuse Treatment*, *131*. https://doi.org/10.1016/j.jsat.2021.108397

Thein, H.-H., Kimber, J., Maher, L., MacDonald, M., & Kaldor, J. M. (2005). Public opinion towards supervised injecting centres and the Sydney Medically Supervised Injecting Centre. *International Journal of Drug Policy*, *16*(4), 275–280. https://doi.org/10.1016/j.drugpo.2005.03.003

Ti, L., Buxton, J., Harrison, S., Dobrer, S., Montaner, J., Wood, E., & Kerr, T. (2015). Willingness to access an in-hospital supervised injection facility among hospitalized people who use illicit drugs. *Journal of Hospital Medicine*, *10*(5), 301–306. https://doi.org/10.1002/jhm.2344

Toth, E. C., Tegner, J., Lauridsen, S., & Kappel, N. (2016). A cross-sectional national survey assessing self-reported drug intake behavior, contact with the primary sector and drug treatment among service users of Danish drug consumption rooms. *Harm Reduction Journal*, *13*(1). https://doi.org/10.1186/s12954-016-0115-0

Tran, V., Reid, S. E., Roxburgh, A., & Day, C. A. (2021). Assessing drug consumption rooms and longer term (5 year) impacts on community and clients. *Risk Management and Healthcare Policy*, *14*, 4639–4647. https://doi.org/10.2147/RMHP.S244720

Trayner, K. M. A., Palmateer, N. E., Hutchinson, S. J., Goldberg, D. J., Shepherd, S. J., Gunson, R. N., Tweed, E. J., Priyadarshi, S., Sumnall, H., Atkinson, A., & McAuley, A. (2021). High willingness to use drug consumption rooms among people who inject drugs in Scotland: findings from a national bio-behavioural survey among people who inject drugs. *International Journal of Drug Policy*, *90*. https://doi.org/10.1016/j.drugpo.2020.102731

Treloar, C., Hull, P., Dore, G. J., & Grebely, J. (2012). Knowledge and barriers associated with assessment and treatment for hepatitis C virus infection among people who inject drugs. *Drug and Alcohol Review*, *31*(7), 918–924. https://doi.org/10.1111/j.1465-3362.2012.00468.x

Treloar, C., Laybutt, B., Jauncey, M., van Beek, I., Lodge, M., Malpas, G., & Carruthers, S. (2008). Broadening discussions of “safe” in hepatitis C prevention: A close-up of swabbing in an analysis of video recordings of injecting practice. *International Journal of Drug Policy*, *19*(1), 59–65. https://doi.org/10.1016/j.drugpo.2007.01.005

Tupper, K. W., McCrae, K., Garber, I., Lysyshyn, M., & Wood, E. (2018). Initial results of a drug checking pilot program to detect fentanyl adulteration in a Canadian setting. *Drug and Alcohol Dependence*, *190*, 242–245. https://doi.org/10.1016/j.drugalcdep.2018.06.020

Tweed, E. J., Rodgers, M., Priyadarshi, S., & Crighton, E. (2018). “Taking away the chaos”: A health needs assessment for people who inject drugs in public places in Glasgow, Scotland. *BMC Public Health*, *18*(1). https://doi.org/10.1186/s12889-018-5718-9

Tyndall, M. W., Kerr, T., Zhang, R., King, E., Montaner, J. G., & Wood, E. (2006). Attendance, drug use patterns, and referrals made from North America’s first supervised injection facility. *Drug and Alcohol Dependence*, *83*(3), 193–198. https://doi.org/10.1016/j.drugalcdep.2005.11.011

Tyndall, M. W., Wood, E., Zhang, R., Lai, C., Montaner, J. S. G., & Kerr, T. (2006). HIV seroprevalence among participants at a Supervised Injection Facility in Vancouver, Canada: Implications for prevention, care and treatment. *Harm Reduction Journal*, *3*. https://doi.org/10.1186/1477-7517-3-36

Urbanik, M. M., & Greene, C. (2021). Operational and contextual barriers to accessing supervised consumption services in two Canadian cities. *International Journal of Drug Policy*, *88*, 102991. https://doi.org/10.1016/j.drugpo.2020.102991

Urbanik, M. M., Maier, K., & Greene, C. (2022). A qualitative comparison of how people who use drugs’ perceptions and experiences of policing affect supervised consumption services access in two cities. *International Journal of Drug Policy*, *104*, 103671. https://doi.org/10.1016/j.drugpo.2022.103671

Valencia, J., Troya, J., Lazarus, J. V., Cuevas, G., Alvaro-Meca, A., Torres, J., Gardeta, C., Lozano, D., Moreno, S., & Ryan, P. (2021). Recurring Severe Injection-Related Infections in People Who Inject Drugs and the Need for Safe Injection Sites in Madrid, Spain. *Open Forum Infectious Diseases*, *8*(7). https://doi.org/10.1093/ofid/ofab251

Van Beek, I., & Gilmour, S. (2000). Preference to have used a medically supervised injecting centre among injecting drug users in Kings Cross, Sydney. *Australian and New Zealand Journal of Public Health*, *24*(5), 540–542. https://doi.org/10.1111/j.1467-842X.2000.tb00507.x

Van Beek, I., Kimber, J., Dakin, A., & Gilmour, S. (2004). The Sydney Medically Supervised Injecting Centre: Reducing harm associated with heroin overdose. *Critical Public Health*, *14*(4), 391–406. https://doi.org/10.1080/09581590400027528

Van Den Boom, W., del Mar Quiroga, M., Fetene, D. M., Agius, P. A., Higgs, P. G., Maher, L., Hickman, M., Stoové, M. A., & Dietze, P. M. (2021). The Melbourne safe injecting room attracted people most in need of its service. *American Journal of Preventive Medicine*, *61*(2), 217–224. https://doi.org/10.1016/j.amepre.2021.02.018

Van Der Poel, A., Barendregt, C., & Van De Mheen, D. (2003). Drug consumption rooms in Rotterdam: An explorative description. *European Addiction Research*, *9*(2), 94–100. https://doi.org/10.1159/000068807

Van Der Poel, A., Barendregt, C., & Van De Mheen, D. (2006). Drug users’ participation in addiction care: Different groups do different things. *Journal of Psychoactive Drugs*, *38*(2), 123–132. https://doi.org/10.1080/02791072.2006.10399836

Vipler, S., Hayashi, K., Milloy, M.-J., Wood, E., Nosova, E., Kerr, T., & Ti, L. (2018). Use of withdrawal management services among people who use illicit drugs in Vancouver, Canada. *Substance Abuse: Treatment, Prevention, and Policy*, *13*(1). https://doi.org/10.1186/s13011-018-0164-3

Wallace, B., Pagan, F., & Pauly, B. B. (2019). The implementation of overdose prevention sites as a novel and nimble response during an illegal drug overdose public health emergency. *International Journal of Drug Policy*, *66*, 64–72. https://doi.org/10.1016/j.drugpo.2019.01.017

Watson, T., Bayoumi, A., Kolla, G., Penn, R., Fischer, B., Luce, J., & Strike, C. (2012). Police Perceptions of Supervised Consumption Sites (SCSs): A Qualitative Study. *Substance Use and Misuse* *47*(4), 364–374. https://doi.org/10.3109/10826084.2011.645104

Watson, T. M., Barnaby, L., Bayoumi, A. M., Challacombe, L., Wright, A., & Strike, C. (2021). ‘This is a health service. Leave it alone’: service user and staff views on policing boundaries involving supervised consumption services. *Addiction Research & Theory*, *29*(1), 55–63. https://doi.org/10.1080/16066359.2020.1730821

Watson, T. M., Bayoumi, A. M., Hopkins, S., Wright, A., Naraine, R., Khorasheh, T., Challacombe, L., & Strike, C. (2018). Creating and sustaining cooperative relationships between supervised injection services and police: A qualitative interview study of international stakeholders. *International Journal of Drug Policy*, *61*, 1–6. https://doi.org/10.1016/j.drugpo.2018.08.001

Watson, T. M., Strike, C., Kolla, G., Penn, R., & Bayoumi, A. M. (2015). “Drugs dont have age limits”: The challenge of setting age restrictions for supervised injection facilities. *Drugs: Education, Prevention and Policy*, *22*(4), 370–379. https://doi.org/10.3109/09687637.2015.1034239

Watson, T. M., Strike, C., Kolla, G., Penn, R., Jairam, J., Hopkins, S., Luce, J., Degani, N., Millson, P., & Bayoumi, A. M. (2013). Design considerations for supervised consumption facilities (SCFs): Preferences for facilities where people can inject and smoke drugs. *International Journal of Drug Policy*, *24*(2), 156–163. https://doi.org/10.1016/j.drugpo.2012.09.003

Wenger, L. D., Arreola, S. G., & Kral, A. H. (2011). The prospect of implementing a Safer Injection Facility in San Francisco: Perspectives of community stakeholders. *International Journal of Drug Policy*, *22*(3), 239–241. https://doi.org/10.1016/j.drugpo.2011.01.001

Wild, T., Koziel, J., Anderson-Baron, J., Asbridge, M., Belle-Isle, L., Dell, C., Elliott, R., Hathaway, A., MacPherson, D., McBride, K., Pauly, B., Strike, C., Galovan, A., & Hyshka, E. (2021). Public support for harm reduction: A population survey of Canadian adults. *PLOS ONE*, *16*(5). https://doi.org/10.1371/journal.pone.0251860

Wodak, A., Symonds, A., & Richmond, R. (2003). The Role of Civil Disobedience in Drug Policy Reform: How an Illegal Safer Injection Room Led to a Sanctioned, ‘Medically Supervised Injection Center. Available from: <https://www.ojp.gov/ncjrs/virtual-library/abstracts/role-civil-disobedience-drug-policy-reform-how-illegal-safer>

Wolf, J., Linssen, L., & De Graaf, I. (2003). Drug consumption facilities in the Netherlands. *Journal of Drug Issues*, *33*(3), 649–661. https://doi.org/10.1177/002204260303300307

Wolfson-Stofko, B., Curtis, R., Fuentes, F., Manchess, E., Del Rio-Cumba, A., & Bennett, A. S. (2016). The Portapotty Experiment: Neoliberal approaches to the intertwined epidemics of opioid-related overdose and HIV/HCV, and why we need cultural anthropologists in the South Bronx. *Dialectical Anthropology*, *40*(4), 395–410. https://doi.org/10.1007/s10624-016-9443-4

Wolfson-Stofko, B., Elliott, L., Bennett, A. S., Curtis, R., & Gwadz, M. (2018). Perspectives on supervised injection facilities among service industry employees in New York City: A qualitative exploration. *International Journal of Drug Policy*, *62*, 67–73. https://doi.org/10.1016/j.drugpo.2018.08.016

Wood, E., Kerr, T., Lloyd-Smith, E., Buchner, C., Marsh, D. C., Montaner, J. S. G., & Tyndall, M. W. (2004). Methodology for evaluating Insite: Canada’s first medically supervised safer injection facility for injection drug users. *Harm Reduction Journal*, *1*. https://doi.org/10.1186/1477-7517-1-9

Wood, E., Kerr, T., Small, W., Li, K., Marsh, D. C., Montaner, J. S. G., & Tyndall, M. W. (2004). Changes in public order after the opening of a medically supervised safer injecting facility for illicit injection drug users. *CMAJ. Canadian Medical Association Journal*, *171*(7), 731–734. https://doi.org/10.1503/cmaj.1040774

Wood, E., Kerr, T., Spittal, P. M., Li, K., Small, W., Tyndall, M. W., Hogg, R. S., O’Shaughnessy, M. V., & Schechter, M. T. (2003). The potential public health and community impacts of safer injecting facilities: Evidence from a cohort of injection drug users. *Journal of Acquired Immune Deficiency Syndromes*, *32*(1), 2–8. https://doi.org/10.1097/00126334-200301010-00002

Wood, E., Kerr, T., Stoltz, J., Qui, Z., Zhang, R., Montaner, J. S. G., & Tyndall, M. W. (2005). Prevalence and correlates of hepatitis C infection among users of North America’s first medically supervised safer injection facility. *Public Health*, *119*(12), 1111–1115. https://doi.org/10.1016/j.puhe.2005.05.006

Wood, E., Tyndall, M. W., Lai, C., Montaner, J. S. G., & Kerr, T. (2006). Impact of a medically supervised safer injecting facility on drug dealing and other drug-related crime. *Substance Abuse: Treatment, Prevention, and Policy*, *1*(1). https://doi.org/10.1186/1747-597X-1-13

Wood, E., Tyndall, M. W., Li, K., Lloyd-Smith, E., Small, W., Montaner, J. S. G., & Kerr, T. (2005). Do supervised injecting facilities attract higher-risk injection drug users? *American Journal of Preventive Medicine*, *29*(2), 126–130. https://doi.org/10.1016/j.amepre.2005.04.011

Wood, E., Tyndall, M. W., Montaner, J. S., & Kerr, T. (2006). Summary of findings from the evaluation of a pilot medically supervised safer injecting facility. *CMAJ. Canadian Medical Association Journal*, *175*(11), 1399–1404. https://doi.org/10.1503/cmaj.060863

Wood, E., Tyndall, M. W., Qui, Z., Zhang, R., Montaner, J. S. G., & Kerr, T. (2006). Service uptake and characteristics of injection drug users utilizing North America’s first medically supervised safer injecting facility. *American Journal of Public Health*, *96*(5), 770–773. https://doi.org/10.2105/AJPH.2004.057828

Wood, E., Tyndall, M. W., Stoltz, J.-A., Small, W., Lloyd-Smith, E., Zhang, R., Montaner, J. S. G., & Kerr, T. (2005). Factors associated with syringe sharing among users of a medically supervised safer injecting facility. *American Journal of Infectious Diseases*, *1*(1). https://doi.org/10.3844/ajidsp.2005.50.54

Wood, E., Tyndall, M. W., Stoltz, J.-A., Small, W., Zhang, R., O’Connell, J., Montaner, J. S. G., & Kerr, T. (2005). Safer injecting education for HIV prevention within a medically supervised safer injecting facility. *International Journal of Drug Policy*, *16*(4), 281–284. https://doi.org/10.1016/j.drugpo.2005.07.004

Wood, E., Tyndall, M. W., Zhang, R., Montaner, J. S. G., & Kerr, T. (2007). Rate of detoxification service use and its impact among a cohort of supervised injecting facility users. *Addiction*, *102*(6), 916–919. https://doi.org/10.1111/j.1360-0443.2007.01818.x

Wood, R. A., Wood, E., Lai, C., Tyndall, M. W., Montaner, J. S. G., & Kerr, T. (2008). Nurse-delivered safer injection education among a cohort of injection drug users: Evidence from the evaluation of Vancouver’s supervised injection facility. *International Journal of Drug Policy*, *19*(3), 183–188. https://doi.org/10.1016/j.drugpo.2008.01.003

Wright, N. M. J., & Tompkins, C. N. E. (2006a). A review of the evidence for the effectiveness of primary prevention interventions for Hepatitis C among injecting drug users. *Harm Reduction Journal*, *3*. https://doi.org/10.1186/1477-7517-3-27

Wright, N. M. J., & Tompkins, C. N. E. (2006b). How can health services effectively meet the health needs of homeless people? *British Journal of General Practice*, *56*(525), 286–293. https://www.scopus.com/inward/record.uri?eid=2-s2.0-33645734219&partnerID=40&md5=19a4d21bfccb2d5c98cd092dc7f36723

Wright, S. (2019). Designing the Debate: Assessing the Role of Design Practices in Safe Injection Sites. Available from: <https://harmreduction.eu/resources/dcr/designing-the-debate-assessing-the-role-of-design-practices-in-safe-injection-sites/>

Xavier, J., Lowe, L., & Rodrigues, S. (2021). Access to and Safety for Women at Supervised Consumption Services. Available from: <https://cmha.ca/wp-content/uploads/2021/04/Women-and-SCS-Report_FINAL-April-2021.pdf>

Yoon, G. H., Levengood, T. W., Davoust, M. J., Ogden, S. N., Kral, A. H., Cahill, S. R., & Bazzi, A. R. (2022). Implementation and sustainability of safe consumption sites: a qualitative systematic review and thematic synthesis. *Harm Reduction Journal*, *19*(1). https://doi.org/10.1186/s12954-022-00655-z

Zampini, G. F. (2014). Governance versus government: Drug consumption rooms in Australia and the UK. *International Journal of Drug Policy*, *25*(5), 978–984. https://doi.org/10.1016/j.drugpo.2014.03.006

Ziegler, B. R., Wray, A. J., & Luginaah, I. (2019). The ever-changing narrative: Supervised injection site policy making in Ontario, Canada. *International Journal of Drug Policy*, *74*, 98–111. https://doi.org/10.1016/j.drugpo.2019.09.006

Zlotorzynska, M., Milloy, M.-J. S., Richardson, L., Nguyen, P., Montaner, J. S., Wood, E., & Kerr, T. (2014). Timing of income assistance payment and overdose patterns at a Canadian supervised injection facility. *International Journal of Drug Policy*, *25*(4), 736–739. https://doi.org/10.1016/j.drugpo.2014.03.014

Zobel, F., & Dubois-Arber, F. (2004). Short appraisal of the role and usefulness of drug consumption facilities (DCF) in the reduction of drug-related problems in Switzerland. Available from: <https://www.semanticscholar.org/paper/Short-appraisal-of-the-role-and-usefulness-of-drug-Zobel-Dubois-arber/cc808138e32b9113de8bfddfdd286e01a43b6cf9>

Zolopa, C., Brothers, T. D., Leclerc, P., Mary, J. F., Morissette, C., Bruneau, J., Hyshka, E., Martin, N. K., & Larney, S. (2022). Changes in supervised consumption site use and emergency interventions in Montreal, Canada in the first twelve months of the COVID-19 pandemic: An interrupted time series study. *International Journal of Drug Policy*, *110*, 103894. https://doi.org/10.1016/j.drugpo.2022.103894

Zurhold, H., Degkwitz, P., Verthein, U., & Haasen, C. (2003). Drug consumption rooms in Hamburg, Germany: Evaluation of the effects on harm reduction and the reduction of public nuisance. *Journal of Drug Issues*, *33*(3), 663–688. https://doi.org/10.1177/002204260303300308
