# Supplementary figures and images for: Overdose prevention centres as spaces of safety, trust and inclusion: A causal pathway based on a realist review
Source: Drug Alcohol Rev. Author manuscript; Available in PMC 2025 Jul 29. (PMC7617959; doi:10.1111/dar.13908)

**Appendix 1: Initial program theory of overdose prevention centres**


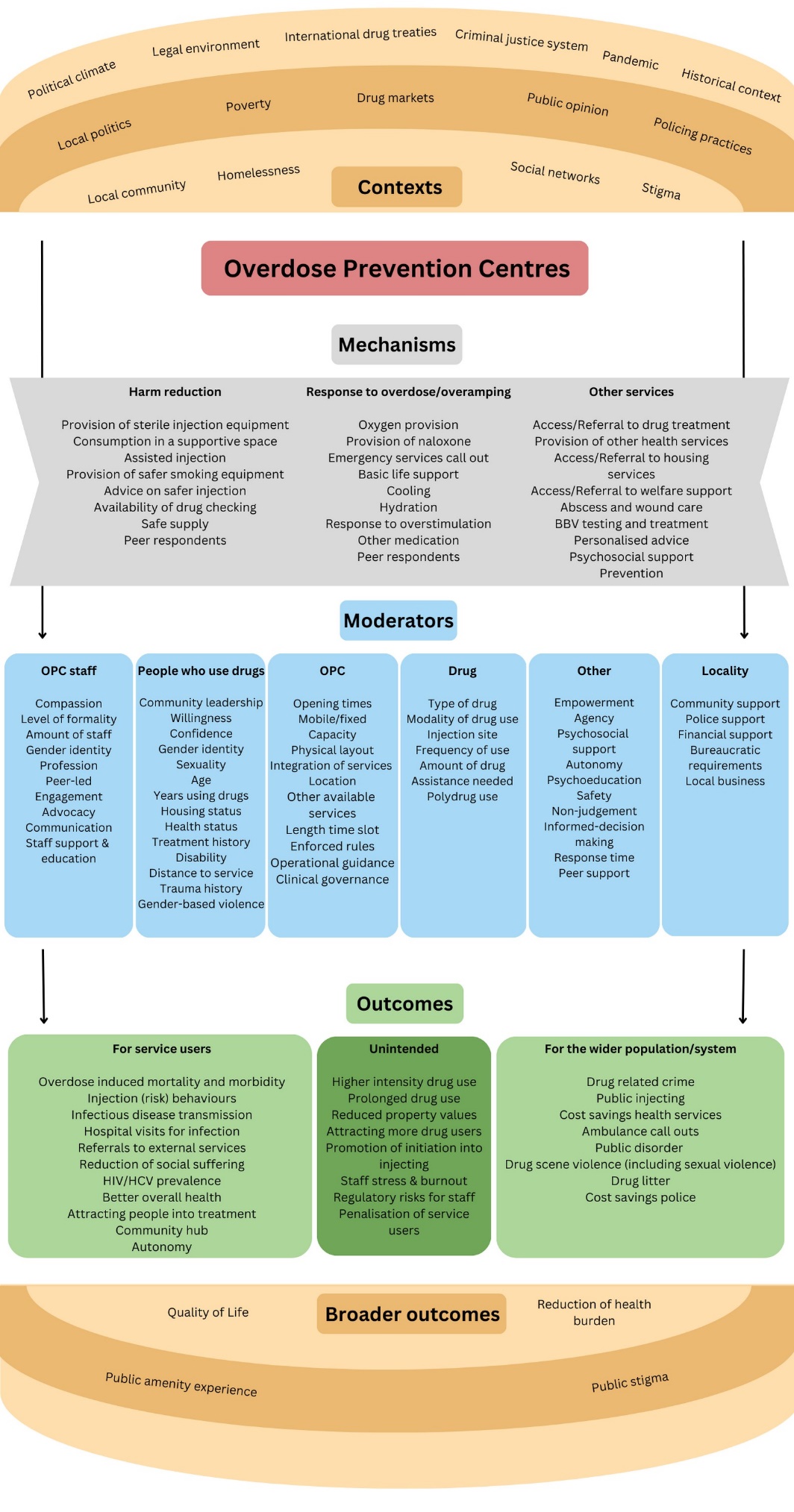

Supplement: Supplementary Material [file EMS206922-supplement-Supplementary_Material.zip › dar13908-sup-0001-appendix1.docx]
